# Antibody reveals conformational latch regulating dimerization in β- and γ-herpesvirus proteases

**DOI:** 10.1038/s41467-026-75438-2

**Published:** 2026-07-13

**Authors:** Marcell A. Zimanyi, Kaitlin R. Hulce, Markus-Frederik Bohn, Jordan Norman, Peter J. Rohweder, Tyler C. Detomasi, Yifan Cheng, Charles S. Craik

**Affiliations:** 1 Department of Pharmaceutical Chemistry, University of California, San Francisco, CA USA; 2 Chemistry and Chemical Biology Graduate Program, University of California, San Francisco, CA USA; 3 Medical Scientist Training Program, University of California, San Francisco, CA USA; 4 Department of Biochemistry and Biophysics, University of California, San Francisco, CA USA; 5 Howard Hughes Medical Institute, University of California, San Francisco, CA USA

**Keywords:** Proteases, Cryoelectron microscopy, Structural biology, Biophysics, Biochemistry

## Abstract

Human herpesvirus (HHV) replication depends on the HHV protease (HHV Pr), an enzyme essential for capsid maturation. Because HHV Pr must transition from an inactive monomer to an active dimer, disrupting dimerization is a promising antiviral strategy. We isolate Fab5, a conformationally selective antibody from a naïve Fab-phage library that recognizes monomeric human cytomegalovirus protease (HCMV) Pr. A 2.6 Å cryo-EM structure reveals Fab5 binds a “latch loop” distal to the active site and dimer interface that secures the C-terminal tail in dimers. Structure-guided mutagenesis in both HCMV Pr and Kaposi’s Sarcoma-associated herpesvirus (KSHV) Pr confirms the functional importance of a 3-residue motif present in β- and γ-HHV Pr latch loops, validating the mechanistic role of the latch loop in dimerization and activity. Because the latch loop is structurally conserved in all HHV Prs, the cryptic sites they form present an avenue for allosteric inhibitor development.

## Abstract (web summary)

Herpesvirus replication depends on a viral protease that must dimerize to become active. Here, authors discover an inhibitory antibody that reveals a structural latch regulating dimerization, identifying a regulatory element across the viral family.

## Introduction

Herpesviruses infect over 90% of the human population and establish lifelong latent infections with periodic reactivation^1,2^. Human cytomegalovirus (HCMV) is particularly problematic, causing devastating disease in immunocompromised patients and being the leading global cause of non-genetic congenital malformations, such as deafness and blindness^3,4^. Current treatments suffer from poor efficacy and toxicity, while the emergence of treatment-resistant viruses underscores the urgent need for therapeutic approaches^5,6^.

Each human herpesvirus (HHV) expresses a serine protease that is essential for viral replication^7,8^. The HHV protease (HHV Pr) activates within the procapsid to cleave scaffolding proteins, leading to capsid maturation (Supplementary Fig. S1). We and others have validated HHV Pr as a therapeutic target by demonstrating that small molecule inhibitors disrupt viral infectivity^9–12^. However, no HHV Pr-targeting compound has advanced through clinical trials. Inhibitor development efforts to date have focused on the active site, which is inherently challenging because it is polar, dynamic and shallow^9^. An orthogonal approach to HHV Pr inhibition is to disrupt the conformational changes required for its activation^5,11^.

**Fig. 1:**
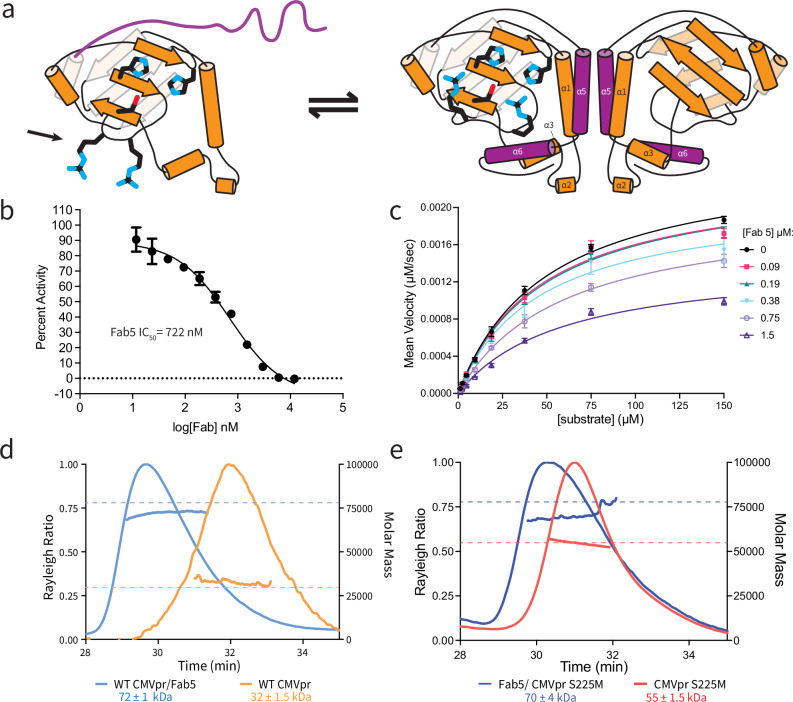
Fab5 is a conformationally selective HCMV Pr inhibitor. **a** Topology map of HCMV Pr monomer and dimer. In the monomeric state, the C-terminal region (purple) remains disordered, and the oxyanion hole loop (OHL, indicated by arrow) is uncoordinated. Upon dimerization, the C-terminal region adopts a defined structure consisting of helices α5 and α6, with an independent active site on each monomer. **b** Fab5 inhibits HCMV Pr activity with an IC_50_ of 722 nM. Data are represented as a mean value ± SD; *n* = 3 technical replicates per concentration point. The IC_50_ was determined by fitting a four-parameter log(inhibitor) vs. response equation. **c** Steady-state kinetics of HCMV Pr performed with increasing concentrations of Fab5. Data are presented as mean values ± SD; *n* = 3 technical replicates per substrate concentration. Kinetic parameters were determined by fitting the Michaelis-Menten equation. **d**, **e** SEC-MALS analysis showing that Fab5 stabilizes the monomeric form of wild-type HCMV Pr. Dashed lines in panels (**d**) and (**e**) indicate expected masses of each protein species. **e** SEC-MALS analysis demonstrating that Fab5 also isolates the dimer-promoting mutant S225M HCMV Pr in a monomeric state. Source data are provided as a Source Data file.

All HHV Prs are structurally and functionally conserved and must form homodimers to achieve catalytic activity^13,14^. The micromolar-range dimerization affinities of HHV Prs provide spatial control of proteolysis during viral replication, allowing for dimerization only within the capsid at high local concentration^15^. As a monomer, the HHV Pr C-terminal region is disordered^16–18^. During dimerization, the C-terminus folds into two major helices, one that forms the dimer interface (helix α5) and another which packs beneath the active site (helix α6). This disorder-to-order transition stabilizes the oxyanion hole loop (OHL), which, together with the Ser-His-His catalytic triad, forms an independent active site on each monomer (Fig. 1a)^13^. While previous studies demonstrated that the disorder-to-order transition occurs, the molecular mechanisms controlling these rearrangements have remained unclear.

Inhibitory compounds often serve as excellent tools for revealing molecular mechanisms. We previously identified small molecule inhibitors that mimic the chemical properties of helix α5 to disrupt dimerization and decrease viral infectivity in cells, but developing high-potency leads has proven challenging^5,11,18^. We therefore sought to expand our repertoire of HHV Pr inhibitors by developing new approaches to dimer disruption. Recombinant antibodies are well suited for this task because they have been used to discover mechanisms of protease inactivation, including non-canonical substrate pocket binding, oligomerization disruption, and allosteric site modulation^19–22^. Antibodies that stabilize monomeric HHV Pr can also shed light on the molecular mechanisms underpinning the disorder-to-order transition to better understand how dimerization leads to catalytic activity.

Here, we describe the discovery of an antibody fragment of antigen binding (Fab) termed Fab5 that inhibits HCMV Pr by preventing dimerization through an allosteric mechanism. High-resolution cryoelectron microscopy (cryo-EM) reveals that Fab5 binds a cryptic site distal from the active site and dimer interface, sequestering a latch loop that normally secures the C-terminal tail during the activation process. This latch loop is structurally conserved across HHV Prs, and a critical 3-residue motif is present in six of nine HHV family members spanning the β- and γ-HHV Prs^11,13,14,23–26^. Mutagenesis of this motif in both HCMV Pr and KSHV Pr disrupts dimerization and neutralizes activity, revealing a conserved regulatory mechanism that can be targeted for antiviral intervention.

## Results

### Fab5 allosterically inhibits HCMV Pr by shifting the monomer-dimer equilibrium

Using a phage-displayed naïve human B-cell derived Fab library, we identified five Fabs that bind HCMV Pr (Fig. 1b, Supplementary Fig. S2). We immobilized HCMV Pr on magnetic beads at low protein concentration to bias binders toward the monomeric species^27,28^. To monitor in vitro HCMV Pr activity in the presence of each Fab, we used a peptide substrate with an internally quenched fluorophore. We selected our best inhibitor, Fab5 (IC_50_ = 722 nM, Fig. 1b), for further study. Fab5 binds HCMV Pr with a K_d_ of 1.6 μM as measured by biolayer interferometry (BLI, Supplementary Fig. S3a).

We performed steady-state kinetics assays of HCMV Pr in the presence of increasing concentrations of Fab5 and fit the results to the Michaelis-Menten equation (Fig. 1c). The mechanism of Fab5 inhibition was evaluated using a Lineweaver-Burk plot (double-reciprocal plot) (Supplementary Fig. S3c); as Fab5 concentration increases, the V_max_ of the reaction decreases and the K_m_ increases modestly (Supplementary Fig. S3d), indicating that Fab5 does not compete with substrate at the active site. This kinetic profile is consistent with mixed-type kinetics previously observed for dimer-disrupting compounds targeting HCMV Pr, suggesting that Fab5 may act allosterically by disrupting dimerization^25^.

To test this hypothesis directly, we used Size-Exclusion Chromatography/Multi-Angle Light Scattering (SEC/MALS) to determine the stoichiometry of the Fab5-HCMV Pr complex. Wild-type HCMV Pr mixed with Fab5 eluted as a single peak with a molecular weight consistent with a 1:1 Fab5:HCMV Pr monomer complex (Fig. 1d). To confirm that Fab5 isolates monomers rather than binding dimers, we repeated the experiment with HCMV Pr S225M, a mutant that strongly promotes dimer formation and exists primarily as a dimer in solution^29^. Despite the stronger dimerization propensity of HCMV Pr S225M, Fab5 sequesters it as a monomer, with SEC/MALS showing a single peak consistent with the 1:1 complex (Fig. 1e).

These results demonstrate that Fab5 selectively binds and isolates HCMV Pr monomers. The Fab5 binding affinity (K_d _= 1.6 μM, measured by BLI in the absence of glycerol) is approximately 44-fold higher than the dimerization K_d_ of HCMV Pr under matched buffer conditions (~ 70 μM in saline buffer), and remains favorable in steady-state assay conditions (10% glycerol, ~ 3.6-fold higher vs. dimerization K_d_ of ~ 5.8 μM)^13^. As Fab5 binds available monomers, it depletes the monomer pool and shifts the equilibrium away from dimer formation. This explains why Fab5 also sequesters the dimer-promoting S225M mutant into monomeric complexes and suggests that Fab5 inhibits HCMV Pr by preventing the conformational changes required for HCMV Pr activation.

### Cryo-EM structures of Fab5/HCMV Pr complex reveal a cryptic site

**Fig. 2:**
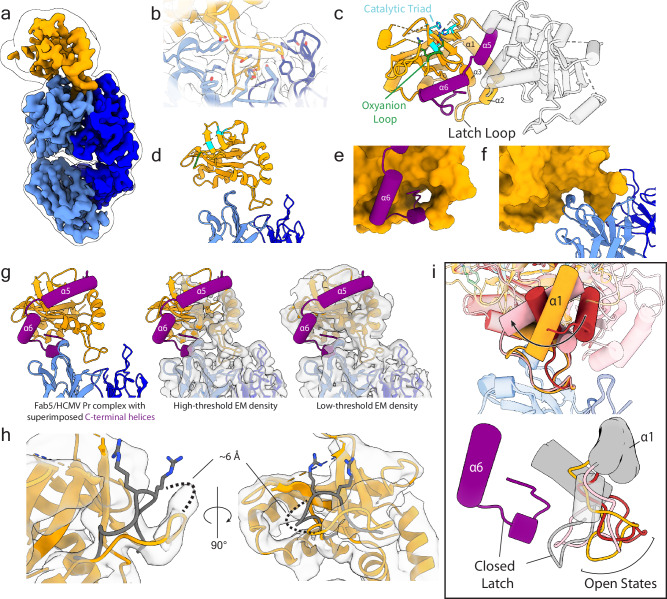
Fab5 binds the HCMV Pr latch loop. **a** Cryo-EM density map of the Fab5/HCMV Pr complex. The Fab5 light chain appears in dark blue, the heavy chain in cornflower blue, and HCMV Pr in orange. The outline contour represents EM density at a lower threshold. **b** Close-up view of the Fab5 epitope. Transparent EM density surrounds the atomic model. **c** Reference dimeric crystal structure of HCMV Pr (PDB:1CMV) with labels for the active site elements in cyan and green. **d** Atomic model of the Fab5/HCMV Pr complex highlighting the interface. **e** View of the latch loop (orange surface) in a closed conformation from dimeric HCMV Pr bound to the C-terminus in PDB:1CMV. **f** The latch loop in its open conformation when bound to Fab5. **g** HCMV Pr C-terminal region from reference dimeric crystal structure (purple tube helices) overlaid on the Fab5/HCMV Pr complex. These helices are a reference to indicate where the C-terminal helices would form upon dimerization. EM density is shown at high (second panel) and low (third panel) thresholds. **h** Low-resolution EM density indicating OHL opening with respect to the dimer (gray). The approximate distance is measured from the alpha carbon of residue R165. **i** 3D classification revealed 3 discrete poses of HCMV Pr (colored orange, red, or pink) relative to Fab5. In panel 1, structures are aligned to Fab5 and show helix α1 twisting up to 65° and tilting up to 23°. In panel 2, structures are aligned to PDB:1CMV (gray and purple). The Fab5-bound latch loops show various states of opening compared to the crystal structure.

To provide structural insights into how Fab5 isolates HCMV Pr monomers, we used cryo-EM to examine their interaction. We determined the structure of the Fab5/HCMV Pr complex, where Fab5 and its epitope are resolved to 2.6 Å (Fig. 2a, b and Supplementary Fig. S4). The resolution of the map varies across the complex, with the highest resolution density at the Fab5-HCMV Pr interface and decreasing resolution in regions of HCMV Pr more distal from the interface. Fab5 binds the latch loop, which is positioned distally from both the active site and dimer interface (Fig. 2c). HCMV Pr residues 111-117 comprise the primary epitope, with Fab5 heavy chain complementarity determining regions (CDRs) making additional contacts with the HCMV Pr core (Fig. 2d). When HCMV Pr is dimeric, the latch loop closes on the C-terminal tail and secures it against the core of the protein (Fig. 2e). Fab5 recognizes an open latch loop conformation and displaces the C-terminal tail (Fig. 2f). Importantly, Fab5 does not block the positions where helices α5 and α6 would form in a dimer, yet no EM density corresponding to these helices appears even at low thresholds (Fig. 2g). This suggests that disrupting the interaction between the C-terminal tail and the latch loop is associated with loss of ordered density for the entire C-terminal region, consistent with the known disorder of the HCMV Pr C-terminus in the monomeric state.

The resolution of HCMV Pr density decreases with distance from Fab5, likely due to flexibility between the two components of the complex, with local resolution estimates indicating substantial variation across the map (Supplementary Fig. S6). The deposited models reflect this variable resolution. At the Fab5-HCMV Pr interface, cryo-EM density supports side chain placements of the latch loop and other residues proximal to Fab5. Distal regions with insufficient density for side chain placement are represented as backbone-only scaffolds, and regions lacking density support are omitted. Although the active site is poorly resolved, we can infer the orientation of the OHL from low-resolution density. The OHL appears to be displaced by ~ 6Å relative to the dimer conformation (measured from the α-carbon of R165) (Fig. 2h), consistent with the open OHL conformation found in inhibited KSHV Pr crystal structures, where helix α6 is also absent (Supplementary Fig. S4)^18^. This affirms that proper OHL positioning requires ordering of the C-terminal region achieved through dimerization.

3D classification revealed two additional poses of HCMV Pr relative to Fab5, allowing us to observe the latch loop in various states of opening (Supplementary Figs. S5, S6). When bound to Fab5, HCMV Pr exhibits a considerable range of motion, allowing for 65° of rotational twist and 23° of tilt (Fig. 2i panel 1, Supplementary Movie 1). Aligning these poses to the dimeric HCMV Pr crystal structure reveals how the latch loop adopts conformations ranging from partially to fully open (Fig. 2i panel 2, Supplementary Movie 2). This flexibility supports our hypothesis that the latch loop is a dynamic element that stabilizes the conformational transitions required for protease activation. Disrupting the latch-tail interface is associated with loss of the ordered C-terminal conformation, consistent with a structural relationship in which the latch loop and C-terminal tail stabilize each other’s ordered states. This reveals a structural element required for the conformational transition associated with HCMV Pr activation.

### The latch loop is essential for HCMV Pr dimerization and activity

**Fig. 3:**
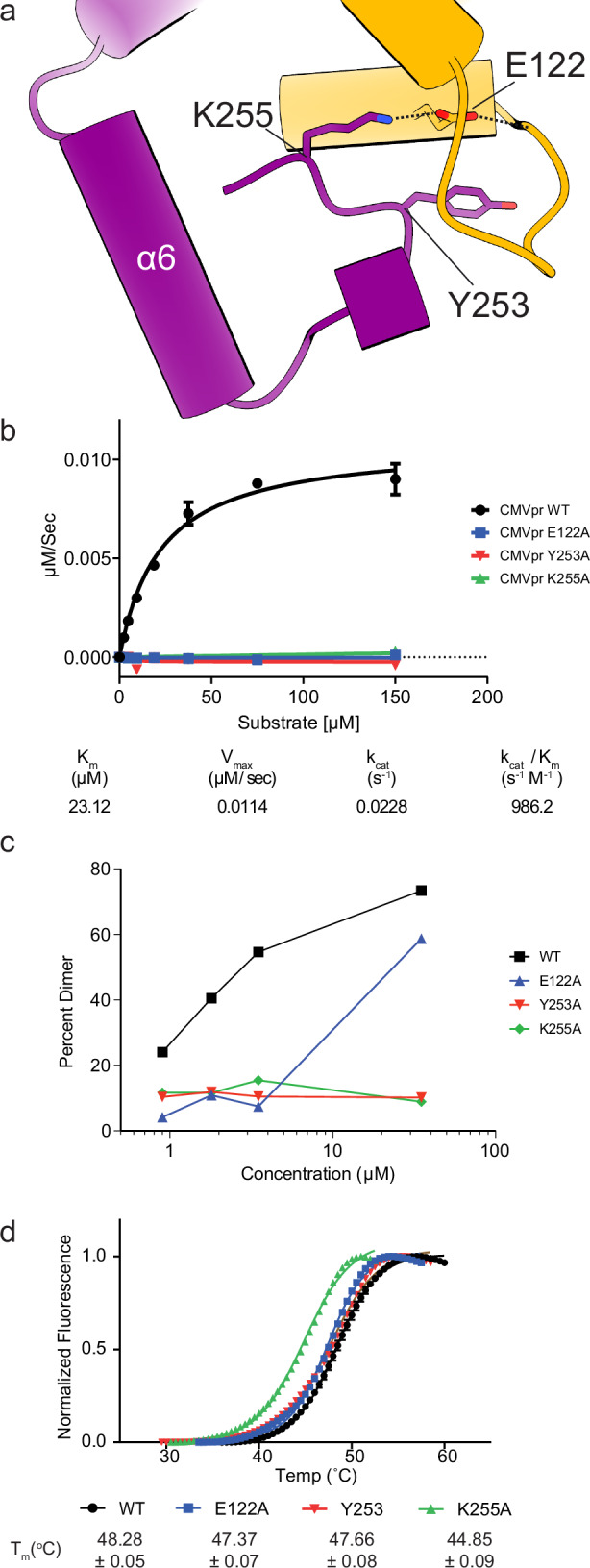
The HCMV Pr latch loop plays an important role in dimerization and activity. **a** Residues E122, Y253, and K255 form critical contacts between the latch loop and the C-terminus and comprise the latch motif. **b** Alanine substitution of any latch motif residue abolishes enzymatic activity as demonstrated by steady-state kinetics assays. Data are presented as mean values ± SD; *n* = 3 technical replicates per substrate concentration. **c** Concentration-dependent dimerization of wild-type HCMV Pr as measured by SEC. Percent dimerization is quantified by the ratio of integrated peak areas corresponding to monomeric and dimeric species, with the trend represented as a line. The X-axis has a log scale with antilog labeling, and all SEC traces can be found in Supplementary Fig. S7. The E122A mutant shows dimerization only at the highest tested concentration, while the Y253A and K255A mutants fail to form dimers at any concentration. **d** Thermal stability profiles of HCMV Pr mutants assessed by DSF. The K255A mutation decreases thermal stability by 3.4 °C compared to wild-type. Other mutations induce shifts of ≤ 1 °C, while the larger K255A shift may reflect local destabilization of the C-terminal region in addition to latch-tail contacts. Source data are provided as a Source Data file.

We investigated the functional role of the latch loop by performing site-directed mutagenesis of key residues and measuring HCMV Pr activity and dimerization. The interface between the latch loop and the HCMV Pr C-terminus features a salt bridge between E122 and K255, while Y253 inserts into a hydrophobic pocket formed by the latch loop with its hydroxyl group remaining solvent-exposed (Fig. 3a). Alanine substitution of any of these three latch motif residues abolishes HCMV Pr enzymatic activity in steady-state kinetics assays (Fig. 3b). We evaluated the effect of these alanine point mutations on dimerization by using SEC, which clearly separates HCMV Pr monomers and dimers into distinct peaks (Supplementary Fig. S7). We quantified the monomer-dimer equilibrium across a concentration range from 0.9 μM to 35 μM by calculating the ratio between the area under the curve of these peaks (Fig. 3c). The E122A mutant only dimerizes at the highest tested concentration (35 μM), while Y253A and K255A mutants remain exclusively monomeric throughout the entire concentration range.

To determine whether these mutations were globally destabilizing or specifically modulating dimerization equilibrium, we measured the thermal stability of the latch mutants using differential scanning fluorimetry (DSF). These experiments were performed at an HCMV Pr concentration of 4 µM, where both monomeric and dimeric species of the wild-type protein would normally be present. The mutants exhibited only modest decreases in apparent melting temperature compared to wild-type HCMV Pr, with K255A showing the largest reduction. Other mutations decreased thermal stability by 1 °C or less (Fig. 3d). These results indicate that the latch mutations do not substantially compromise the overall protein fold or stability, suggesting their effects on protease activity and dimerization are mechanistic rather than structural. The K255A mutation exhibits a notably larger reduction in apparent melting temperature (− 3.4 °C) compared to E122A and Y253A ( ≤ 1 °C), raising the possibility that local destabilization of the C-terminal region may contribute to its loss of activity. Furthermore, as DSF was performed at concentrations well below the HCMV PR dimerization K_d_, these measurements primarily report on monomer thermal stability and do not directly address dimer interface stability. Collectively, our findings establish that the C-terminal latch plays a critical role in regulating both HCMV Pr dimerization and catalytic activity.

### Structural and functional conservation of the latch loop in β- and γ-herpesvirus proteases

**Fig. 4:**
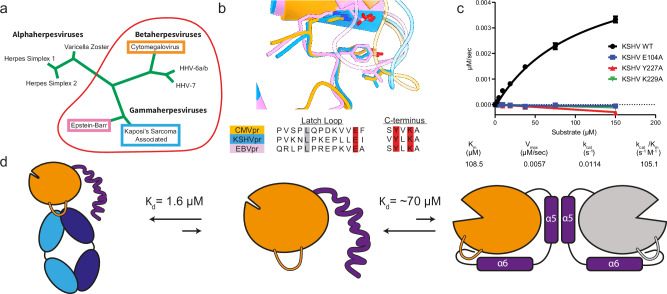
Structural and functional conservation of the latch loop in β- and γ-herpesvirus proteases. **a** Phylogenetic tree of the HHV family. The red circle indicates which HHV Prs have a conserved Latch Motif. **b** Structural superposition and sequence alignment of HCMV Pr, KSHV Pr, and EBV Pr. The alignment demonstrates conservation of the latch loop structure and the latch motif residues across multiple proteases. **c** Functional validation showing that alanine mutations of latch motif residues in KSHV Pr disrupt enzymatic activity in steady-state kinetics assays, similar to the effect observed with HCMV Pr mutants. Data are presented as mean values ± SD; *n* = 3 technical replicates per substrate concentration. (**d**) Mechanistic model illustrating the equilibria between Fab5 and HCMV Pr. Fab5 binds HCMV Pr monomers with higher affinity than the dimerization threshold under all tested buffer conditions, shifting the equilibrium away from dimer formation and towards the Fab5-HCMV Pr complex. By targeting the latch loop, Fab5 prevents dimerization. In the absence of Fab5, the latch loop functions to stabilize the C-terminal region in the active dimeric state of HCMV Pr. Source data are provided as a Source Data file.

The nine HHVs are classified into three subfamilies (α, β, and γ). Though HCMV Pr has a sequence similarity of only 24–38% compared with other HHV Prs, all known HHV Pr structures have high structural conservation, including the latch loop. Despite this sequence divergence, the three-residue HCMV Pr latch motif (E122, Y253, and K255) is conserved in representative β and γ HHV Prs (Fig. 4a). Structural alignment of HCMV Pr, KSHV Pr and Epstein-Barr virus protease (EBV Pr) indicates that the latch motif forms the interface between the latch loop and the C-terminus across these two HHV subfamilies (Fig. 4b). We assessed the conserved role of these residues with alanine substitution of the latch motif in KSHV Pr, which results in an inactivated enzyme as it does in HCMV Pr (Fig. 4c).

The sequence, structure, and functional conservation of the latch loop mechanism in β- and γ-HHV Prs reveals a fundamental mechanism regulating HHV Pr activation that may extend across the HHV family. A bioinformatic analysis using sequence alignment of all sequenced HHV Prs to generate a sequence logo reveals partial conservation of the latch motif across the viral family (Supplementary Fig. S8a). The leucine in the latch loop and the corresponding tyrosine near the C-terminus are fully conserved across α-HHV Prs. The primary variation in α-HHV Prs is a lysine-to-glutamine substitution at the position equivalent to K255 in HCMV Pr. While this substitution replaces a salt bridge with a potential hydrogen bond, this glutamine could still provide a stabilizing interaction with the latch loop (Supplementary Fig. S8c). Structural alignments of existing α-HHV Pr structures confirm that while the latch loop is structurally present, the overall interface between the latch and the C-terminus appears less extensive than in β- and γ-HHV Prs (Supplementary Fig. S8b). It is worth noting that in α-HHV Pr structures, either the C-terminal tail or the latch loop has poor resolution and thus missing residues, which we modeled using AlphaFold for hypothesis generation. This loss of density may indicate flexibility in these regions, which could reflect weaker but still functional latch-tail interactions. Overall, while all HHV Prs share an architectural framework, the energetic contribution of the latch regulatory mechanism may be particularly critical in β- and γ-HHV Prs.

## Discussion

The latch loop regulates dimerization in β- and γ-herpesvirus proteases through a conserved three-residue motif. While previous models have emphasized how dimerization drives ordering of helices α5 and α6, our findings reveal a bidirectional relationship where the latch loop securing the C-terminal tail is essential to complete the disorder-to-order transition. The equilibrium between HHV Pr monomers and dimers depends on this C-terminal ordering, with the latch loop serving as the critical structural element stabilizing the rearrangement (Fig. 4d).

Structural characterization using X-ray crystallography has been a pivotal tool for the discovery of compounds that bind HHV Pr and establishment of the disorder-to-order transition model^11,13,14,23–26^. However, structural information showing this disorder-to-order transition has remained elusive because the high protein concentrations required for crystallization make it difficult to study HHV Pr monomeric conformations and its many flexible loops. Using single-particle cryo-EM, we examined the Fab5/HCMV Pr complex at concentrations 100-fold lower than those required for crystallography and characterized its heterogeneity and flexibility. This work reinforces the enabling role of Fabs as conformationally selective fiducial markers^30^ and demonstrates that cryo-EM can successfully resolve Fab epitopes even for small, flexible targets with micromolar affinity^31^.

The latch loop exemplifies long-range allosteric regulation in enzyme function. By disrupting this peripheral loop, Fab5 destabilizes the active site by preventing the ordering of large structural elements. In addition, entropy may also contribute to the Fab5 inhibitory mechanism. We recently provided comprehensive evidence linking cryo-EM density loss and B-factor changes to conformational flexibility and entropy redistribution in protein complexes^32^. Namely, rigidification of a protein interface can lead to increased flexibility in distal areas. Molecular dynamics studies of homologous KSHV and Varicella Zoster virus (VZV) proteases corroborate this model by indicating that dimerization stabilizes the dimer interface and increases mobility at the substrate binding pocket^33^. Fab5 binding may introduce flexibility in both the active site and the C-terminal region, as indicated by the respective loss of EM density. This general increase in flexibility may be an entropic barrier that prevents helices α5 and α6 from ordering even though Fab5 would sterically permit their rearrangement.

Other allosteric serine protease inhibitors such as the anti-human Kallikrein-related peptidase Fab (anti-hKLK5) highlight how antibodies can be potent and specific by exploiting the mechanisms of inhibition^34^. Fab5 serves as a tool compound demonstrating how the challenges of targeting the HCMV Pr active site can be circumvented via latch loop binding. While Fab5 likely lacks the potency for direct therapeutic application, it validates the latch loop as a druggable target and provides structural blueprints for rational inhibitor design. Beyond small molecules, antibody-based therapeutics targeting HHV Prs are an intriguing possibility. Recent advances in intracellular antibody delivery via electroporation, nanoparticle protein carriers and adeno-associated virus gene transfer are expanding the feasibility of cytoplasmic antibody inhibitors^35–37^. If delivery challenges can be overcome, the conformational selectivity and allosteric mechanism demonstrated by Fab5 could be leveraged for therapeutic development.

While our functional studies are limited to HCMV and KSHV, the sequence and structural conservation of the three-residue latch motif across β- and γ-HHV Prs strongly suggests this regulatory mechanism extends to EBV Pr and other members of these subfamilies. Though latch loop sequences diverge among the HHV Prs, the pocket formed by the latch loop, which normally engages the C-terminal tail, has similar chemical and structural properties across the viral family. This site presents an attractive target for inhibitor development with the potential for cross-reactive inhibition via these shared structural features. This study provides proof-of-principle for structural and functional characterization of Fab-HHV Pr interactions, and similar approaches applied to other HHV Prs could expand inhibitor repertoires and potentially lead to the discovery of cross-reactive antibodies. More broadly, our findings establish the latch loop as a conserved regulatory element that can be exploited to combat HHV replication. The latch loop mechanism highlights how dynamic conformational changes can control enzyme function and underscores the importance of characterizing cryptic sites.

## Methods

### Phage display panning

All Fabs in this study were isolated from a fully human naïve B-cell Fab-phage library (diversity 4.1 × 10^10^)^38^. HCMV Pr was biotinylated using EZ-Link NHS-Chromogenic-Biotin (Pierce) and buffer-exchanged into buffer containing 25 mM potassium phosphate at pH 8.0 and 150 mM KCl (protein buffer, PB). Biotinylated HCMV Pr was immobilized to magnetic streptavidin-coated beads (Invitrogen) blocked with 2% BSA in PB. Four rounds of panning were performed with decreasing amounts of HCMV Pr concentration (100 nM, 50 nM, then 25 nM two times) to progressively enrich for high-affinity binders. In each round of panning, the library was precipitated with PEG/NaCl (20% PEG 8000, 2.5 M NaCl), resuspended in PB with 1% BSA, and incubated with HCMV Pr-coated beads at room temperature for 1.5 h. Negative selection against uncoated magnetic beads was performed before antigen capture rounds in rounds 2-4 to deplete non-specific clones. Beads were then washed in PB with 0.1% Tween 20, with wash stringency increased in each round (10, 15, then 20 washes). Bound phage were eluted with freshly prepared 100 mM triethylamine for 5 min, immediately neutralized with 1 M Tris-HCl pH 7.4, and used to infect log-phase TG1 *E. coli* (Millipore) at 37 ^o^C. Phages were amplified using M13KO7 helper phage in 2xYT medium supplemented with 100 μg/mL ampicillin and 50 μg/mL kanamycin, then re-precipitated with PEG/NaCl. These phages were used as the input into the next round of panning. After the final round, infected TG1 was diluted 1:1000 and plated on 2xYT agar plates with 100 μg/mL ampicillin and 2% glucose, where individual colonies could be observed. Individual clones were screened by inoculating individual colonies into wells of 96-well plates containing 2xYT with 100 μg/mL ampicillin and 2% glucose and grown overnight at 37 ^o^C. The following day, a 96-pin replicator was used to inoculate overnight cultures into 160 μL 2xYT with 100 μg/mL ampicillin and 0.1% glucose and grown to OD_600_ = ~ 0.6. Fab expression was induced through the addition of 40 μL of 2xYT containing 5 mM IPTG and incubated overnight at 30 ^o^C. These plates were then spun down at 2000 x *g*, and the crude supernatant was used for ELISA analysis to identify binders. 50 μL of streptavidin in PB was added to Maxisorp 96-well plates (Nunc) and incubated overnight, washed 2 times in PB, and blocked for 2 h in PB with 2% BSA at room temperature. Wells were washed 3 times in PB, and 100 μL of 1 μM biotinylated HCMV Pr in PB was added to each well and incubated at room temperature for 1 h. Wells were washed 3 times with PB, then the crude supernatant from the overnight expressions were added to each well and incubated for 1 h at room temperature. Wells were washed 3 times with PB with 0.1% Tween 20, and 50 μL of anti-Myc HRP conjugate (Bio-Rad Cat# MCA2200P, RRID:AB_324087) was added to the plate for 1 hour at room temperature. Wells were wash 3 times with PB with 0.1% Tween 20 before the addition of 50 μL of Pierce Turbo TMB to each well. Plates were shaken for 15 min before the addition of 15 μL of 2.5 M H_2_SO_4_. Absorbance was measured at 450 nm on a BioTek Synergy H4 plate reader. Clones with a positive signal were sequenced, and unique clones were expressed and purified for further analysis.

### Fab purification

50 mL of 2 x YT AG media (100 μg/mL ampicillin, 2% glucose) were inoculated with transformed BL21(DE3) E. coli colonies. and the cultures were grown overnight at 30 °C. Starter cultures were diluted to OD600 ∼ 0.05 in 1 L of 2xYT + 0.01% glucose + ampicillin (100 μg/mL) and grown at 37 °C to an OD600 of 0.6. Protein expression was induced with 1 mM IPTG, and the culture was shaken at 20 °C overnight. The periplasmic protein fraction, which contains the expressed Fabs, was isolated via an osmotic shock protocol. Briefly, E. coli cultures were centrifuged at 6500 x *g* for 10 min, and the pellets were resuspended in 20 mL ice-cold TES buffer (0.2 M Tris, pH 8.0, 0.5 mM EDTA, 0.5 M sucrose) and incubated on ice for 15 min before addition of 20 mL of ice-cold MilliQ water supplemented with protease inhibitors (cOmplete protease inhibitor cocktail, EDTA-free, Roche) and gentle rocking for 30 min. Cells were pelleted by centrifugation and supernatants taken for purification via HisPur™ Ni-NTA Resin following the manufacturer’s protocols. Ni-NTA purified Fabs were dialyzed in PB and further purified via size exclusion FPLC on an AKTA autopurification system (General Electric) using a Superdex 200 10/300GL column using an isocratic PBS mobile phase. Fractions were analyzed with SDS-PAGE in reducing and non-reducing conditions, and concentrations determined by absorbance at 280 nm using calculated extinction coefficients (https://web.expasy.org/protparam/).

### HCMV Pr constructs and mutants

All HCMV Pr constructs (UL80, residues 1–256) bear a N-terminal 6X-His tag in addition to the mutations A141V, A143V, P144A and A209V to protect against autoproteolysis. Point mutagenesis for latch motif residues was performed by designing primers with the desired point mutation in the center of the primer and predicted annealing temperatures of 68–72 °C. PCR reactions were prepared by mixing 1 ng of template with 0.5 μM final of primers in ultrapure water and finally 2x Q5 master mix (NEB). PCR was performed by first holding the mixture at 95 °C for 3 min, then 28 cycles of 95 °C for 10 s, 15 s of annealing at various temperatures, and extension at 72 °C for 2.5 min. Final extension was done for 3 minutes at 72 °C. Samples were then digested with DpnI (NEB) for 1 hour at 37 °C, then transformed into DH5-alpha cells (NEB). Cultures were grown in 5 mL Luria Broth (LB) supplemented with ampicillin (100 μg/mL final concentration) while shaking at 37 °C overnight. DNA was isolated using a Qiagen Miniprep Kit. Primers for.

### HHV Pr Purification

HCMV Pr, KSHV Pr (ORF17, residues 1–230), and their respective latch motif mutants were expressed in Rosetta 2 BL21 *E. coli* cells (Millipore). Cells were grown in 50 mL LB supplemented with AMP (100 μg/mL) while shaking at 37 °C overnight. The next day, 10–50 mL of culture was used to inoculate 1L LB supplemented with ampicillin (100 μg/mL) and shaken at 37 °C to an OD600 of 0.6. Isopropyl β-d-1-thiogalactopyranoside (IPTG) was added (1 mM final concentration), and cultures were shaken at 16 °C overnight. Cells were harvested, pelleted and suspended in a buffer containing 50 mM potassium phosphate at pH 8.0, 300 mM KCl, 25 mM imidazole and 5 mM 2-mercaptoethanol (BME). Cells were lysed by microfluidization and pelleted, and the supernatant was purified on a GE HealthCare LifeSciences Akta Explorer FPLC at 4 °C. Protein was eluted over two stacked 5 mL HisTrap Nickel columns with gradient elution into a buffer containing 25 mM potassium phosphate at pH 8.0, 150 mM KCl, 300 mM imidazole and 5 mM BME. Eluate was collected and dialyzed overnight against a buffer containing 25 mM potassium phosphate at pH 8.0, 150 mM KCl, 0.1 mM EDTA and 1 mM BME. Dialyzed protein was concentrated to ~2 mL and purified over HiLoad 26/60 Superdex 75 (GE Healthcare) into the same buffer. Protein bands were analyzed by SDS-PAGE and pure protein was collected, flash frozen and stored at − 80 °C. Protein concentrations were determined using a NanoDrop 2000c UV spectrophotometer (Thermo Scientific) using an extinction coefficient of 28,420 M^−1^ cm^−1^ for all HCMV Pr constructs.

### Steady-state kinetics

HCMV Pr wild-type or mutant was diluted to 500 nM in buffer containing 25 mM potassium phosphate at pH 8.0, 150 mM KCl, 0.1 mM EDTA, 1 mM BME, 10% glycerol and 0.01% TWEEN-20. An internally quenched FRET substrate was used: NH_2_-Lys(MCA)-Tbg-Tbg-Asn-Ala-Ser-Ser-Arg-Leu-Lys(Dnp)-Arg-OH, where Tbg is L-*tert*-leucine, Lys(MCA) is a lysine residue with side chain linked to a 7-methoxycoumarin-4-acetic acid, and Lys(Dnp) is a lysine linked to 2,4-dinitrophenyl. Substrate was prepared in 1:2 serial dilutions from 7.5 – 0.06 mM in DMSO, then 98 μL protein + 2 μL substrate (150 – 0.3 μM final concentration) in a 96-well black untreated polystyrene plate. Enzyme velocity was monitored by fluorescence increase (excitation: 328 nm, emission: 393 nm) on a BioTek Synergy H4 Bioreader at 30 °C. Mean velocity (RFU/s) during steady state was fit using BioTek Gen5 data analysis software. The mean velocity of control wells containing only buffer and substrate was averaged and subtracted from the calculated enzyme velocity. A serial dilution of free MCA was prepared, ex/em at 30 °C was determined, and fluorescence (RFU) was plot in triplicate against MCA concentration (μΜ), then fit to a linear regression, which was used to convert initial velocity values from RFU/s to μM/s. Substrate concentration vs. velocity curves were plot in GraphPad Prism then fit using the standard Michaelis-Menten and *k*_*cat*_ equations then resulting values were used to calculate *k*_*cat*_/K_m_ with standard propagation of error. All data were collected duplicate or triplicate from technical replicates and are reported in figures as the mean, including error bars depicting standard deviation.

### IC_50_ values

HCMV Pr was diluted to 1 μM in buffer containing 25 mM potassium phosphate at pH 8.0, 150 mM KCl, 0.1 mM EDTA and 10% glycerol. Fab 1, 2, 3, 4 or 5 was diluted in 1:3 serial dilution from 8 − 0 μM in phosphate-buffered saline (PBS), then 48 μL HCMV Pr solution and 50 μL Fab solution was combined in a 96-well black untreated polystyrene plate and incubated for 20 min at room temperature (500 nM HCMV Pr final, 5% glycerol). The combination of HCMV Pr solution in 10% glycerol and Fab solution in PBS yields a final glycerol concentration of approximately 5%. A 7-amino-4-carbamoylmethylcoumarin (ACC) cleavable substrate was used: Ac-NH-Tbg-Tbg-N4Me_2_Asn-Ala-ACC, where Ac-NH refers to an acyl capped N-terminus, Tbg is L-*tert-*Leucine, and N4Me_2_Asn is N4,N4-dimethyl asparagine. Substrate diluted in DMSO was added to the HCMV Pr + Fab solution (2 μL, 40 μΜ final) and enzyme velocity was monitored by fluorescence increase (excitation: 380 nm, emission: 460 nm) on a BioTek Synergy H4 Bioreader at 30 °C. The velocity (V) of PBS-only controls was averaged and used to normalize data as percent activity (1):

1 $$\%{{{\rm{Activity}}}}=\,\left(\frac{{{{{\rm{V}}}}}_{{{{\rm{compound}}}}}}{{{{{\rm{V}}}}}_{{{{\rm{DMSO}}}}}}\right)\times 100$$

The log_10_[Fab] was plotted vs. percent activity, and curves were fit with a standard four-parameter log(inhibitor) vs. response with variable slope equation.

### Biolayer-interferometry

HCMV Pr was diluted into PBS, then biotinylated for 1 h at room temperature using EZ-Link NHS-Chromogenic-Biotin (Pierce)^28^. Fab5 was dialyzed into buffer containing 25 mM potassium phosphate at pH 8.0, 150 mM KCl and 0.1 mM EDTA, then diluted to 10,000 nM and serially diluted 2x into 5 more concentrations in the above phosphate buffer plus 1% bovine serum albumin and 0.01% TWEEN-20. All assays were performed without the presence of glycerol. Biotinylated HCMV Pr was immobilized on ForteBio streptavidin SA biosensors at a concentration of 1 μM. All measurements were performed in PB with 1% BSA in 384-well plates. Data were analyzed using a 1:1 interaction model with global fitting on the ForteBio data analysis software (9.0.0.6). KD values were determined by the fitting of either equilibrium or maximum response (nm) as a function of Fab concentration.

### Size-exclusion chromatography and Multi-angle light scattering

Wild-type HCMV Pr, HCMV Pr S225M, or Fab5 mixed with either HCMV Pr at equimolar amounts were prepared in buffer containing 25 mM potassium phosphate, pH 8.0 and 150 mM KCl at a total protein mass of 200 µg. Samples were incubated at room temperature for 1 h before 500 µL of the mixture was injected onto a Superdex75 10/300 column and eluted over 1.2 CV. Multi-angle light scattering was performed by running the elution from an SEC run and injecting into a Wyatt Dawn Pro and Optilab combined instrument. Data were plotted in Astra and imported to GraphPad Prism, where they were overlayed on normalized SEC data using a second Y-axis.

### Size-exclusion chromatography for HCMV Pr dimerization

WT HCMV Pr was diluted (0.9 μM, 1.8 μM, 3.5 μM or 35 μM final concentration) into buffer containing 25 mM potassium phosphate, pH 8.0, 150 mM KCl, 0.1 mM EDTA, 10% glycerol and 1 mM β-mercaptoethanol, then incubated at room temperature for 1 h. A Superdex 75 10/300 GL column was equilibrated at 4 °C into the same buffer used to dilute proteins. For all protein samples, 500 μL was injected onto the column and protein eluted over 1 CV at 0.6 mL/min flow rate while monitoring Absorbance at 280 nm. For each individual sample run, the absorbance at A280 was plotted flow volume (mL). The minimum absorbance value across the full spectrum was calculated in Microsoft Excel and subtracted from each absorbance value to correct the baseline to zero. The maximum absorbance was calculated for each sample, then all data were normalized to this value using the following Eq. (2), where A_x_ is the 280 nm absorbance at time x and A_max_ is the maximum value:

2 $${Normalized}\,{Intensity}=\,\left(\frac{{A}_{x}}{{A}_{\max }}\right)\times 100$$

### Differential scanning fluorimetry

Protein melting temperatures were determined via differential scanning fluorimetry (DSF). DSF measurements were made using a Bio-Rad C1000 qPCR system in FRET mode. Protein (4 µM) was added to 5x SYPRO dye (Invitrogen, S6651) and plated in triplicate in a white, 96-well PCR plate in PBS. The temperature was held at 23 °C for 5 min before slow ramping in 0.5 °C increments every 30 s. Raw data were normalized from 0 to 1 and trimmed past these minimum and maximum values before fitting in Prism. Data were fit to a Boltzmann sigmoid, and midpoints of curves are reported as melting temperature with 95% confidence interval indicated.

### Cryo-EM Sample preparation and data collection

Fab5/HCMV Pr complex was purified by SEC and concentrated to 1 mg/mL. 3 microliters were pipetted onto glow-discharged gold grids coated in holey carbon film (Quantifoil, 300 mesh 1.2/1.3) or holey gold film (UltrAuFoil, 300, mesh 1.2/1.3). Grids were then blotted and plunge-frozen using a Vitrobot Mark IV equipped with Whatman type 4 blotting paper with a blotting time of 4 s and blotting force of − 2, at 4 °C and 100% humidity. Data were collected at the Janelia Research Campus in Ashburn, VA on Krios 3. This 300 keV microscope is equipped with a Thermo Scientific Falcon 4i camera, Selectris X energy filter, and a cold Field emission gun with 6 eV slit width during acquisition. A nominal magnification of 165,000x was used for a physical pixel size of 0.743 (0.371 in super resolution) with a total dose of 50 e-/A ° 2. Automated data collection was performed using SerialEM 4.1 to collect movies with a defocus range between 0.8-1.8 Å.

### Image processing

Dose-fractionated images stacks were motion corrected and Fourier-cropped by 2 using both cryoSPARC v. 4.7.1 and MotionCor2 1.4.5^39^. CTF estimation was performed using cryoSPARC, followed by micrograph curation, blob-based particle picking, 2D classification, and ab initio modeling. The ab initio model was used to generate templates for further particle picking and curation. Iterative rounds of multi-class ab initio modeling and heterogeneous refinement were used to generate particle stacks where HCMV Pr could be resolved in the complex. Particles were then sent to Relion using the PyEM code suite^40^ for 3D Classification. Particles were then reimported to cryoSPARC for further 3D Classification and 3D refinement.

### Model building and refinement

A model of Fab5 generated by Alphafold 3 was docked into cryo-EM density and refined using ISOLDE 1.6 and PHENIX 1.21^41,42^. The density corresponding to Fab5 and its direct interface with HCMV Pr are well-resolved with clear density for sidechain and backbone model building. To identify the

Fab5 epitope, we generated each flexible loop of HCMV Pr as a peptide in Coot 0.9.6^43^ and systematically fit them into the non-Fab5 cryo-EM density. We identified that the loop from P111-P117 fit this density well and then began building the HCMV Pr residues de novo. After building about 20 residues with high confidence, we used rigid body fitting to append the rest of an HCMV Pr monomer onto it (PDB:1CMV) using well-resolved flanking secondary structural elements as landmarks and proceeded with further refinement using PHENIX. The deposited atomic models distinguish two categories of HCMV Pr coordinates. Residues are modeled where cryo-EM density can strongly support the placement of side chain atoms, and regions where cryo-EM resolution is present but insufficient to support side chains are retained as backbone-only reference scaffold. The complete model was further refined using real-space refinement in PHENIX and ISOLDE. Final collection, refinement and validation statistics are reported in Supplementary Table 1.

### Sequence logo analysis

A sequence similarity network (SSN) was generated using the BLAST option for the following sequence of CMV protease “MTMDEQQSQAVAPVYVGGFLARYDQSPDEAELLLPRDVVEHWLHAQGQGQPSLSVALPLNINHDDTAVVGHVAAMQSVRDGLFCLGCVTSPRFLEIVRRASEKSELVSRGPVSPLQPDKVVEFLSGSYAGLSLSSRRCDDVEAATSLSGSETTPFKHVALCSVGRRRGTLAVYGRDPEWVTQRFPDLTAADRDGLRAQWQRCGSTAVDASGDPFRSDSYGLLGNSVDALYIRERLPKLRYDKQLVGVTERESYVKA” using the EFI-EST tool with default options^44^. The resulting search retrieved 530 sequences, and SSN was generated using an alignment score of 53, which binned the sequences into 3 groups that correspond to Beta gamma and alpha herpesvirus families that was checked by manual curation. A multiple sequence alignment (MSA) was generated using the separated sequences for each of the three families in Clustal Omega^45^, and then a sequence logo was generated using weblogo 3^46^ with the height set to 6 bits to compare across families.

### Reporting summary

Further information on research design is available in the Nature Portfolio Reporting Summary linked to this article.

## Supplementary information

Supplementary information

Description of Additional Supplementary File

Supplementary Movie 1

Supplementary Movie 2

Reporting Summary

Transparent Peer Review file

## Source data

Source data

## Data Availability

The cryo-EM maps have been deposited in the Electron Microscopy Data Bank (EMDB) under accession codes EMD-72658 (Fab5/HCMV Pr complex, class 1); EMD-72659 (Fab5/HCMV Pr complex, class 2); and EMD-72660 (Fab5/HCMV Pr complex, class 3). The atomic coordinates have been deposited in the Protein Data Bank (PDB) under accession codes 9Y7L (Fab5/HCMV Pr complex, class 1); 9Y7M (Fab5/HCMV Pr complex, class 2); and 9Y7N (Fab5/HCMV Pr complex, class 3). Raw cryo-EM micrographs and particle stacks have been deposited in the Electron Microscopy Public Image Archive (EMPIAR) under accession code EMPIAR-13274. The previously published structure referenced in this study is available under accession code 1CMV. Source data are provided in this paper.
