# Supplementary material for: Antibody reveals conformational latch regulating dimerization in β- and γ-herpesvirus proteases
Source: Nat Commun. 2026 Jul 13;17:8590. doi: 10.1038/s41467-026-75438-2 (PMC13486704; doi:10.1038/s41467-026-75438-2)
Supplement: Supplementary file 1 [file 41467_2026_75438_MOESM1_ESM.pdf]

Supplementary Information

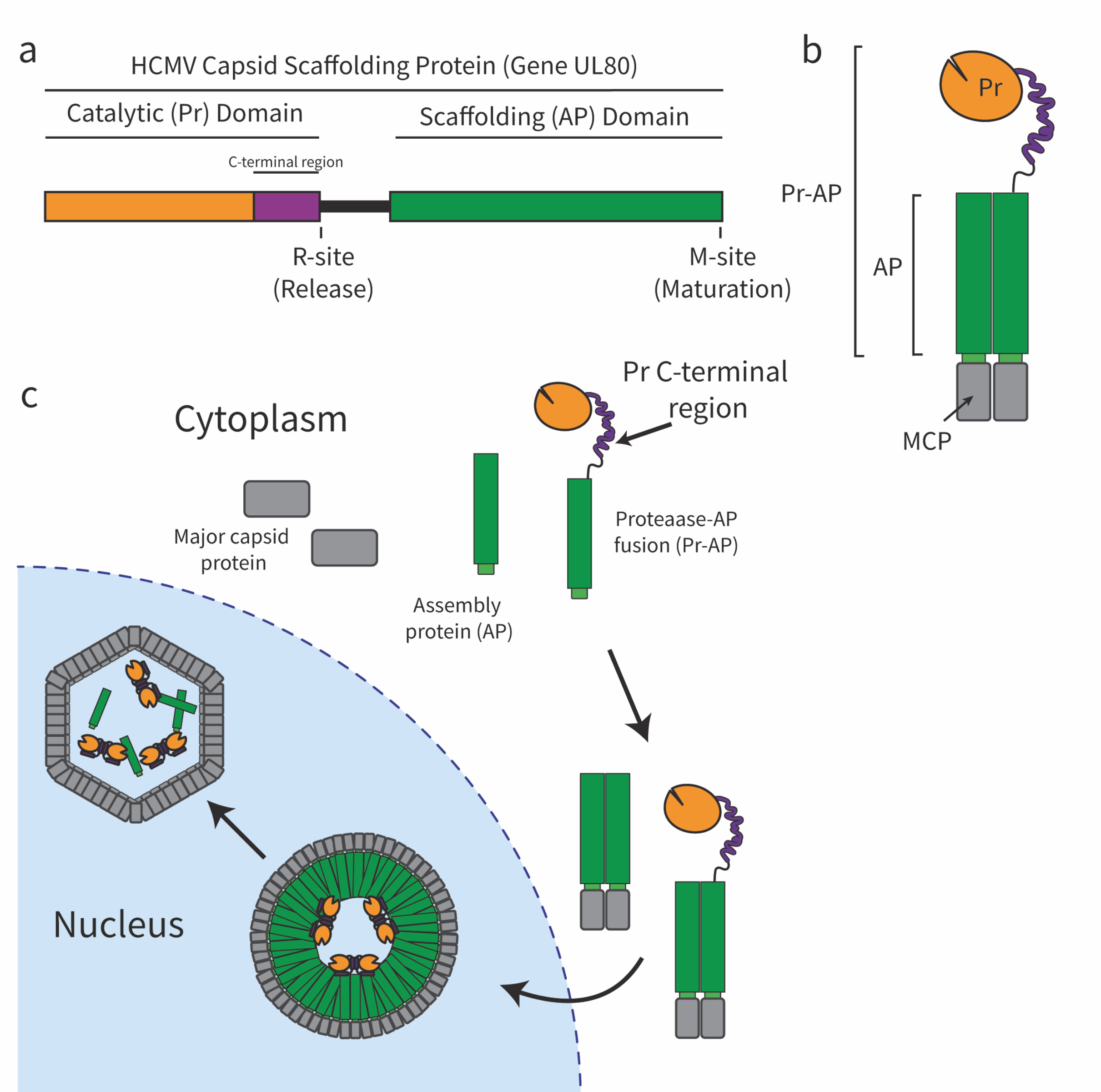

Figure S1: HCMV Pr gene and function (a-b) The HCMV UL80 gene. Primary components are the catalytic domain, a linker region, and the assembly protein (AP) Scaffolding domain. Proteins can either be expressed as Pr-AP or AP due to multiple open reading frames. (c) Pr-AP associates with the major capsid protein (MCP) in the cytosol. Catalytically competent Pr frees itself from AP via the R-site, and the scaffolding is cleared away via the M-site. The wild-type Pr domain contains multiple autoproteolytic sites, which is why our expression construct contains mutations (A141V, A143V, P144A and A209V) to limit autoproteolysis.

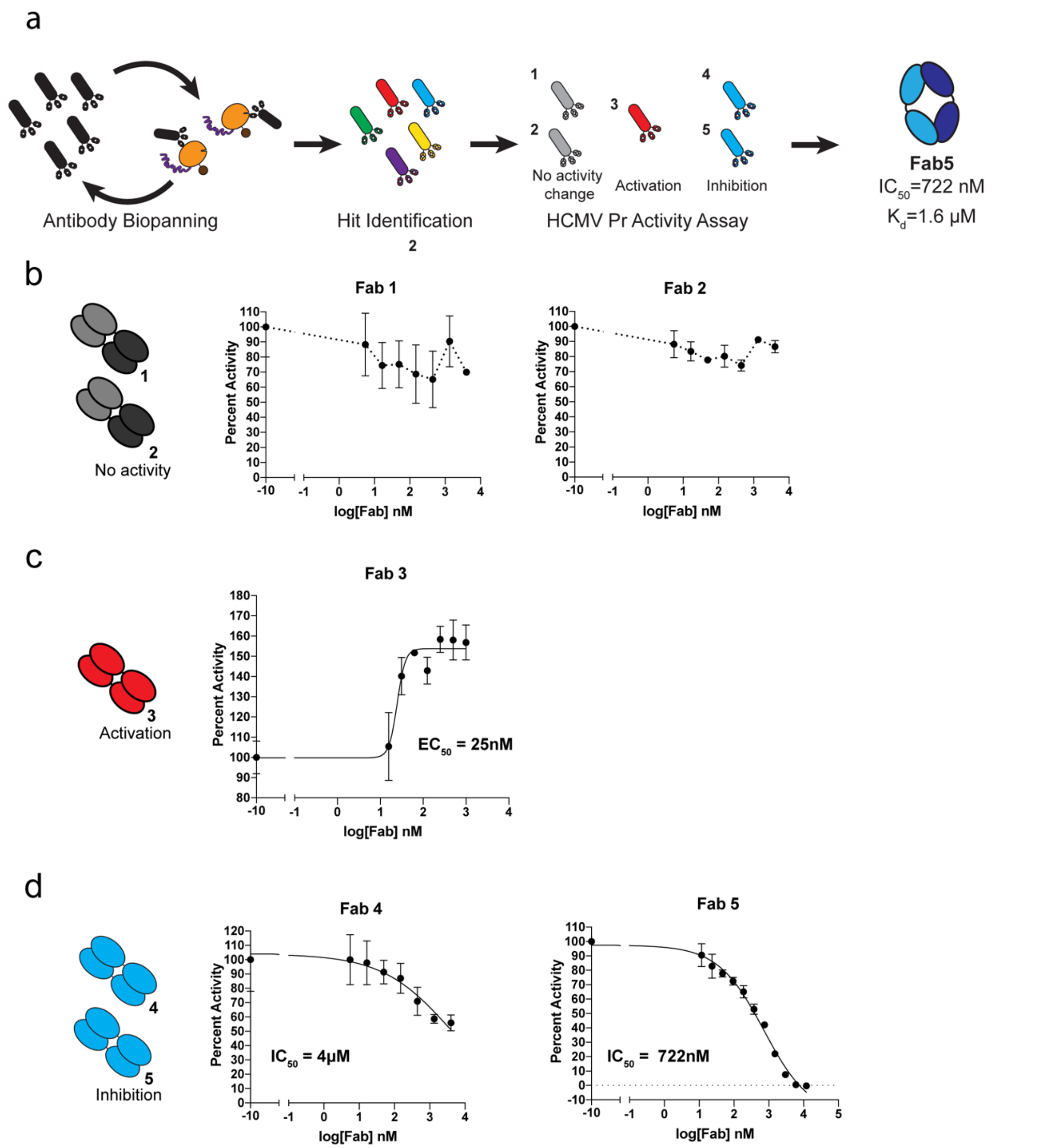

Figure S2: Inhibition assay with the five unique clones from HCMV Pr phage-displayed panning (a) a schematic of the panning campaign against HCMV Pr. Five hits were expressed and purified then tested for inhibition of HCMV Pr activity. (b) Fabs 1 and 2 do not affect HCMV Pr activity. (c) Fab3 has an activating effect on HCMV Pr activity. (d) Fabs 4 and 5 inhibit HCMV Pr. Data are represented as a mean value ± SD; n=3 technical replicates per concentration point. The IC50 was determined by fitting a four-parameter log(inhibitor) vs. response equation. Source data are provided as a Source Data file.

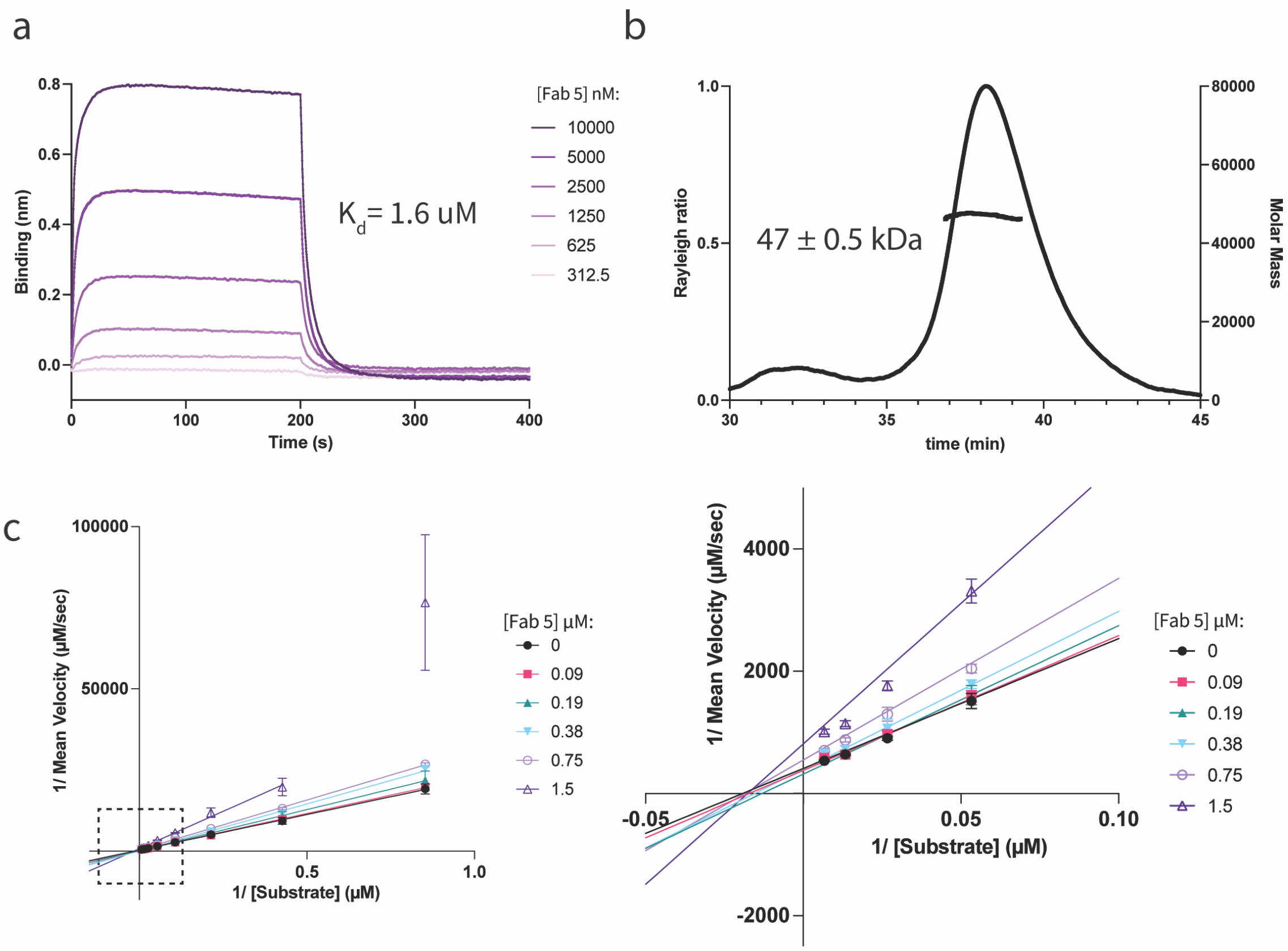

d

[Fab5] μM	K_m (μM)	V_{max} (μM/sec)
0	50.5 ± 5.1	0.00254 ± 0.0001
0.09	47 ± 8.2	0.00235 ± 0.0002
0.19	49.1 ± 7.8	0.00237 ± 0.0002
0.38	46 ± 11.7	0.00210 ± 0.0002
0.75	58.7 ± 7.5	0.00199 ± 0.0001
1.5	59.36 ± 11.8	0.00144 ± 0.0001

Figure S3: Fab5 BLI, SEC/MALS, and Steady-State Kinetics Assays (a) Biolayer interferometry sensogram of Fab5 binding to immobilized HCMV Pr. The dissociation constant (K_d) was determined by fitting the steady-state response to a 1:1 equilibrium binding model. (b) SEC/MALS experiment with Fab5 alone using an S200 10/300 SEC column. (c) Double-reciprocal plot of steady-state kinetics assay measuring HCMV Pr activity performed with Fab5 at various concentrations. Full data is plotted in panel 1, and a magnified view of the dotted line-boxed region is plotted in panel 2. The outlier from the 1.5 μM dataset which had the lowest substrate concentration is excluded from the linear regression. (d) The K_m and V_{max} of HCMV Pr steady-state kinetics are provided in a table for each concentration of Fab5. Data are presented as mean values ± SD; n=3 technical replicates per substrate concentration. Source data are provided as a Source Data file.

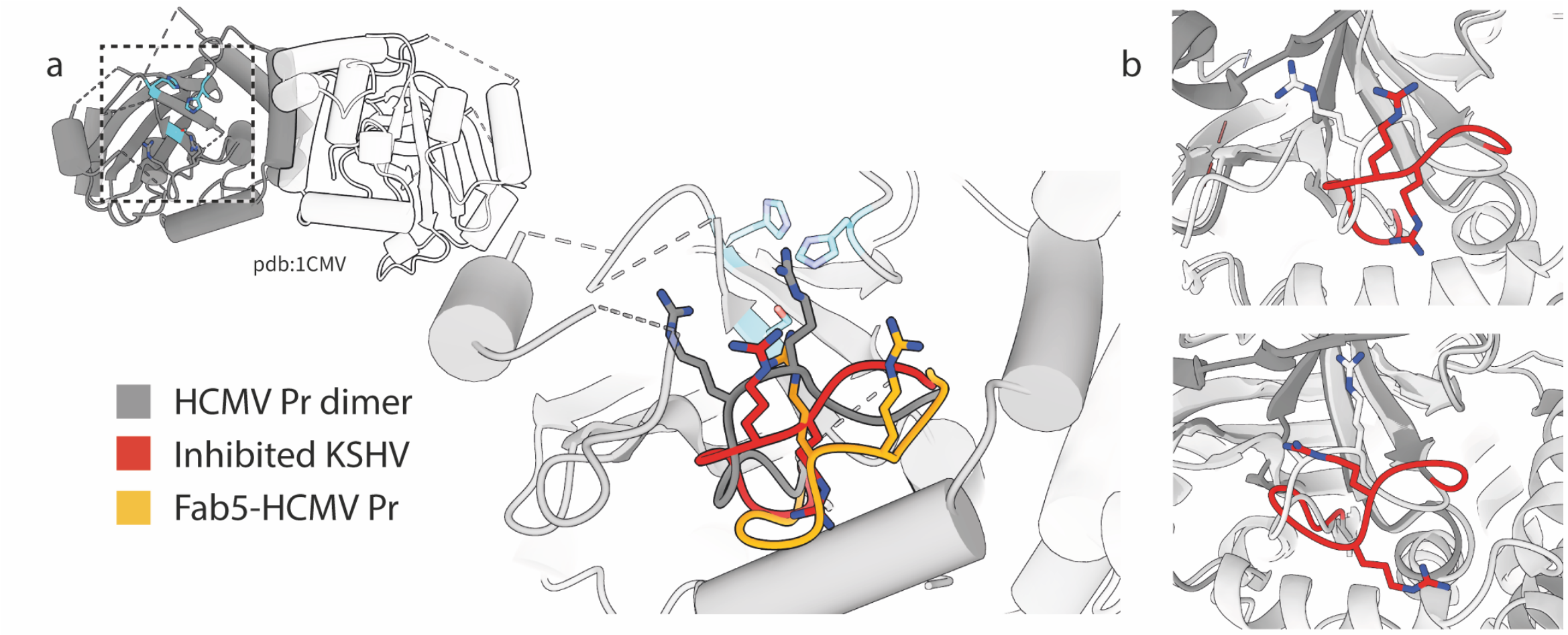

Figure S4: Structural comparison of the OHL (a) HCMV Pr (gray structure) with active site residues colored cyan. The three loops in the foreground are OHL loops from an HCMV Pr dimer (gray, PDB:1CMV), inhibited KSHV Pr (red, PDB:3NJQ Gene ORF17), and the Fab5-HCMV Pr complex (orange). (b) Comparison of KSHV latch loops between dimeric active KSHV (light gray) and inhibited KSHV (red). The biological assembly of inhibited KSHV Pr in the crystal structure PDB:3NJQ is dimeric despite the deletion of the C-terminal region. The inhibitor, which mimics helix α5 and displaces it, forms a crystal contact between 2 monomers. The OHL assumes different conformations in chains A and B (red), however both OHL conformations are more open compared to the wild-type structure (light gray, PDB:1FL1) and the highlighted arginines point away from the catalytic triad.

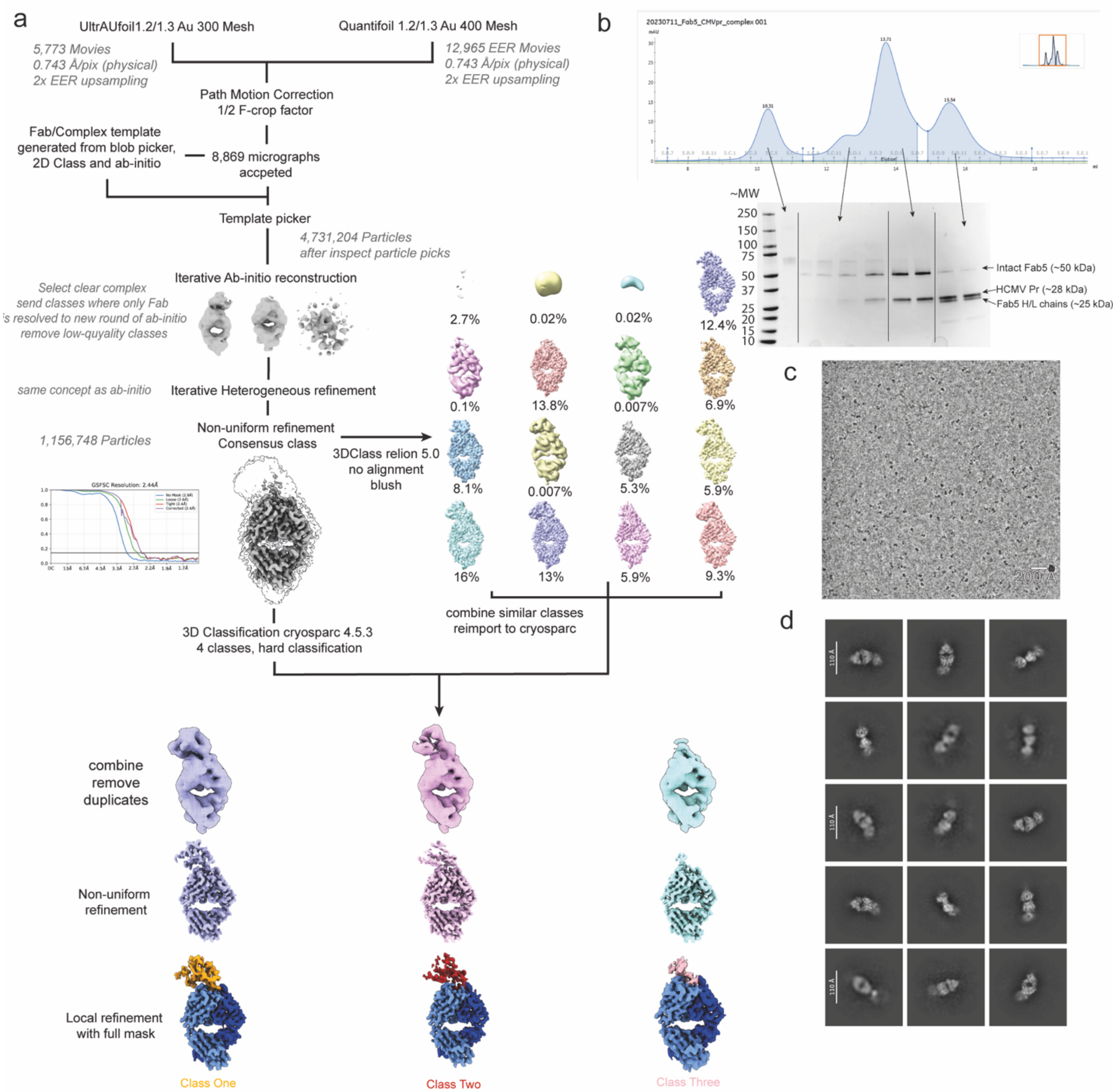

Figure S5: Cryo-EM Data Processing Workflow (a) Flow chart of EM processing workflow. Final volumes for classes One, Two, and Three were solved using 3D classification, non-uniform refinement, and local refinement with a full mask. All volumes were used iteratively for model building. (b) size-exclusion chromatography of Fab5/HCMV Pr complex preparation. Only the largest peak was used for cryo-EM sample. (c) Representative micrograph of Fab5/HCMV Pr complex. (d) Representative 2D classes of Fab5/HCMV Pr complex.

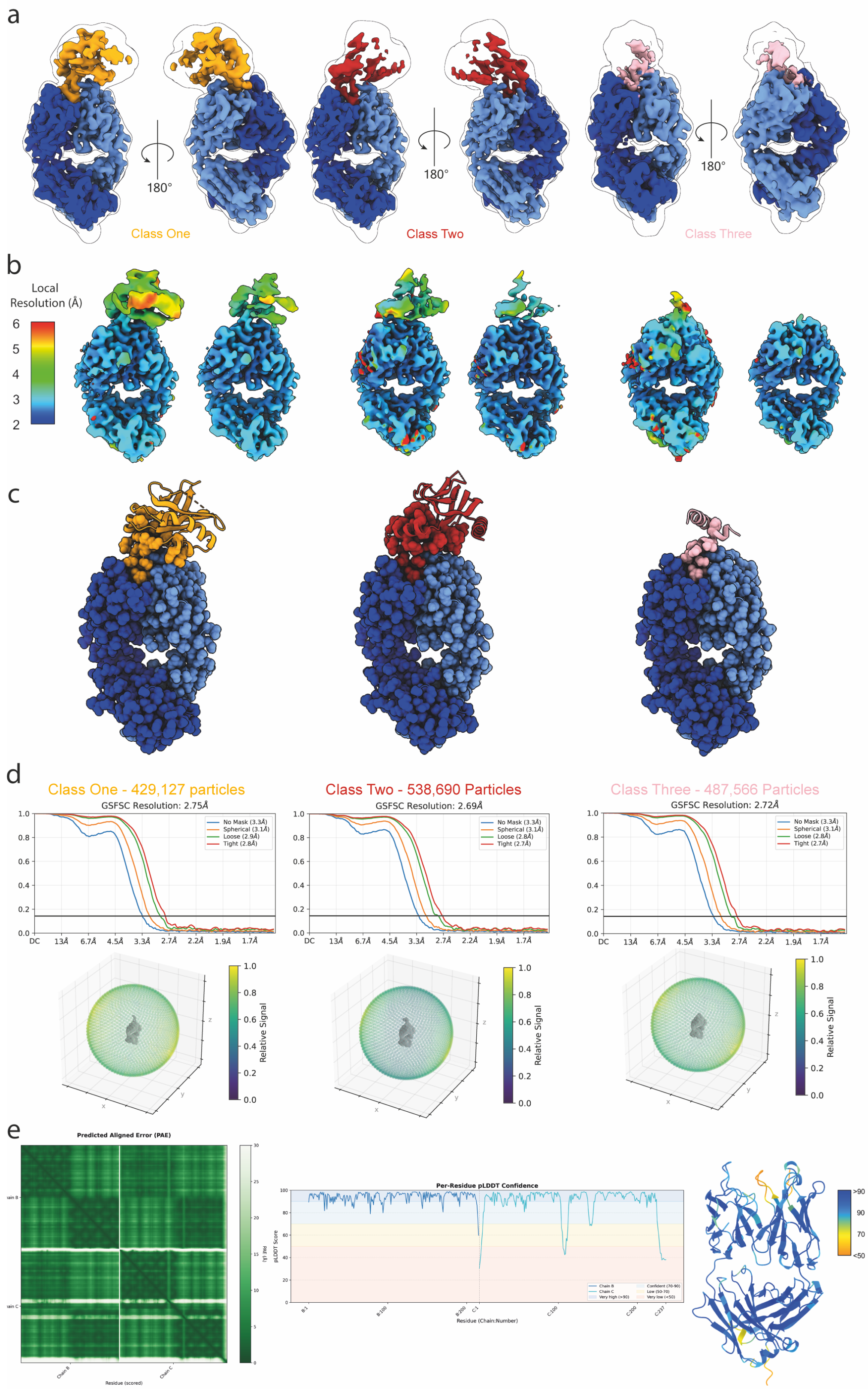

Figure S6: Cryo-EM Map Quality and Models (a) For each class, two views of the map are provided with an outline of the density at a low threshold. (b) local resolution is estimated for each class and depicted

on maps with front and back views at 2 different thresholds. (c) Atomic models for all classes, with atoms depicted as spheres and backbone-only reference scaffold shown as cartoons. (d) Fourier shell correlation (FSC) curves for each volume and orientation diagnostics where relative signal is shown relative to viewing angle. e) Alphafold 3 confidence statistics for the Fab5 initial model are shown. The first graph is predicted aligned error (PAE) graphed per residue then the predicted local difference test (pLDDT) scores per residue are graphed and depicted on the initial model.

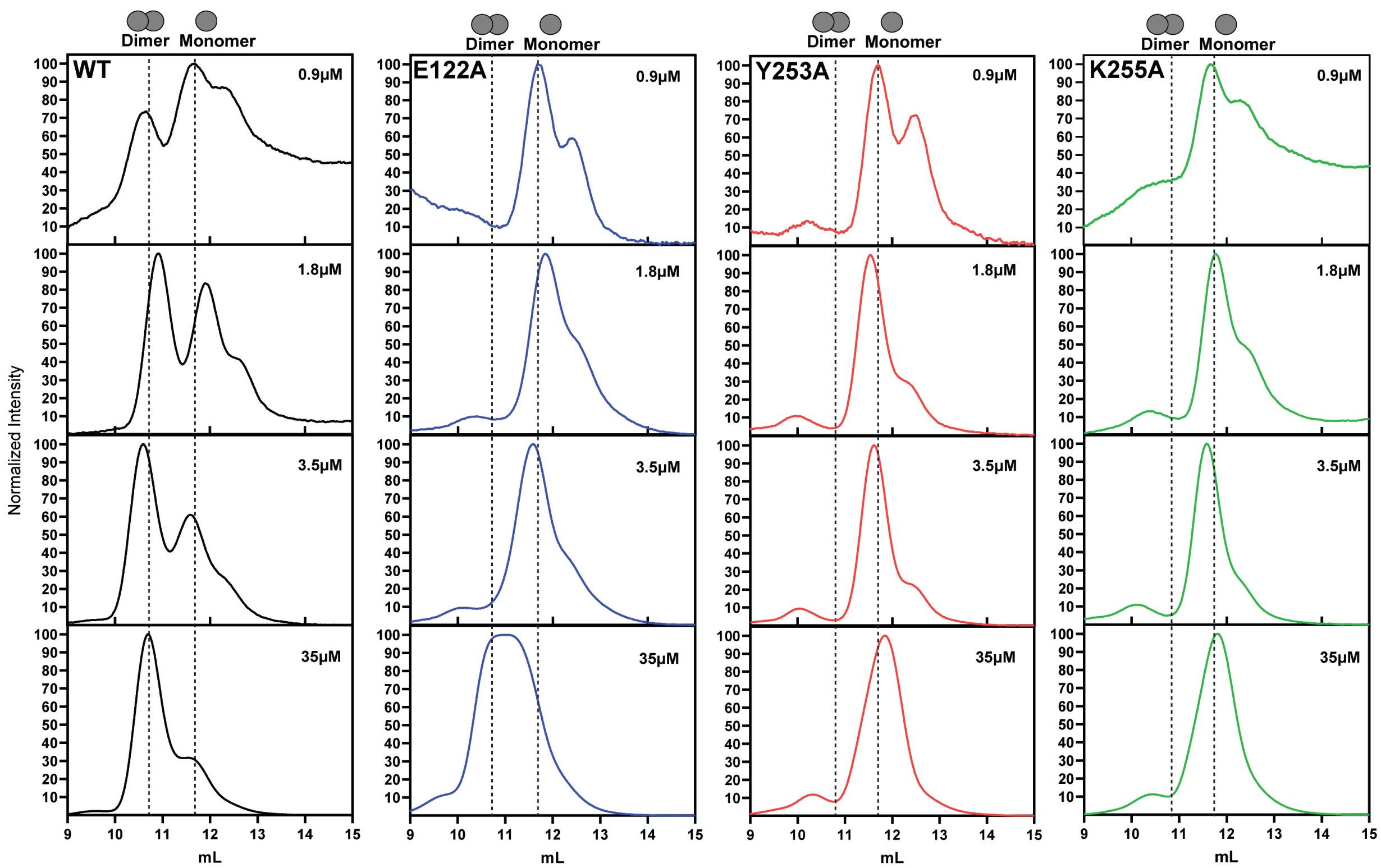

Figure S7: All SEC chromatograms from dimerization experiments. Peak areas were integrated, and a ratio was taken to make the percent dimer graph in Fig. 3c. Results are normalized for intensity, and the x-axis has column volume. The left column is wild-type HCMV Pr, and concentration-dependent dimerization can be observed. All mutant constructs remain as monomers except for E122A, which can dimerize at the highest concentration. Source data are provided as a Source Data file.

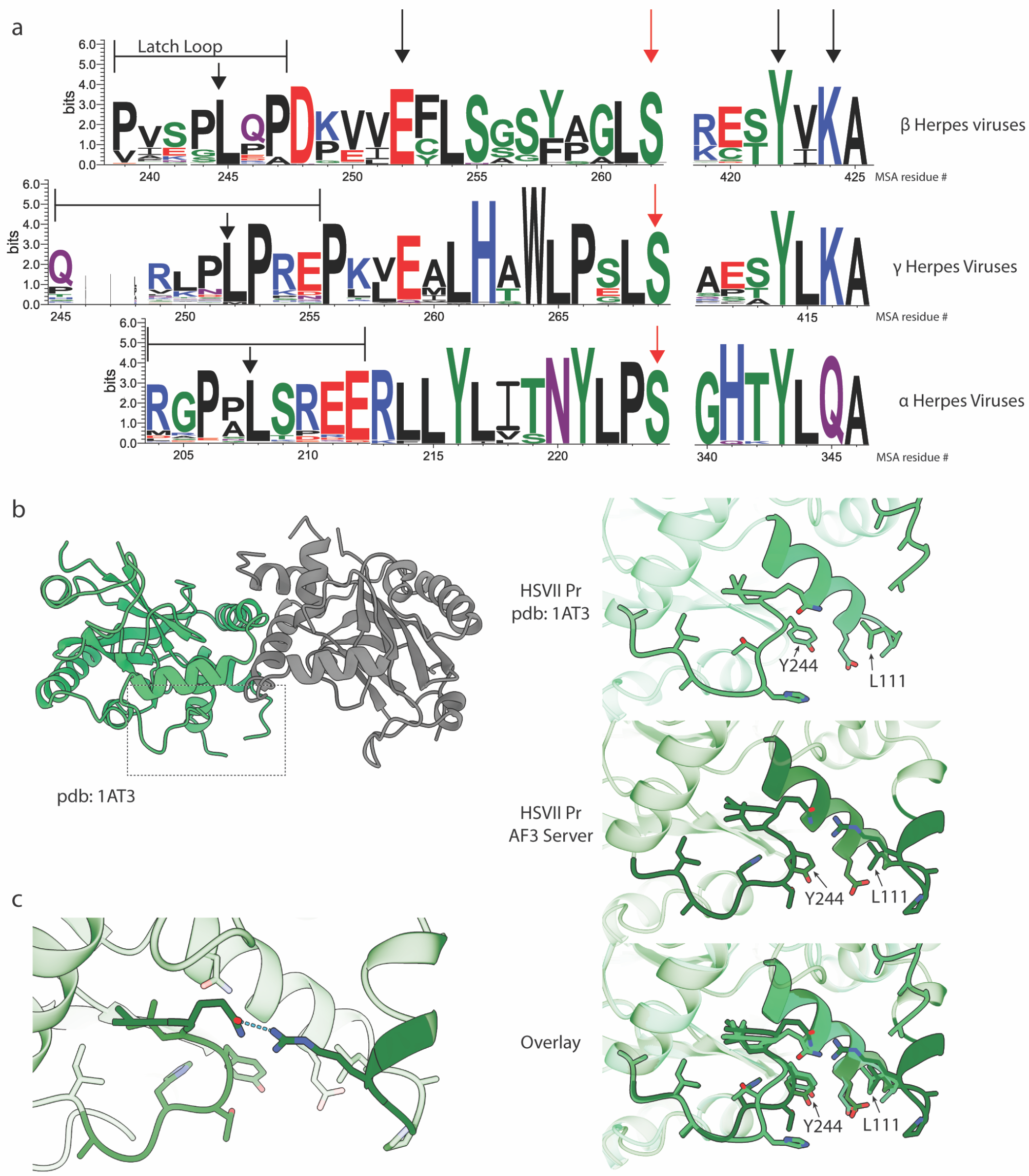

Figure S8: Multiple sequence alignment across HHV subfamilies and the αHHV Pr latch. (a) Sequence logos that were generated by constructing an MSA of all sequences in UniprotKB corresponding to beta, gamma and alpha herpes virus proteases. The region containing the latch loop and the active-site S and the c terminus of the protein are depicted, and functionally equivalent positions are vertically aligned. The residues important for the latch loop binding are denoted with a black arrow and the active site serine is demarcated with a red arrow. While the register does shift between families the Leucine in the latch loop is highly conserved along with its partner the Y. The K and E pair is only conserved in the gamma and beta viruses demonstrating the sequence conservation of this element. (b) Structural views of the αHHV Pr latch represented by HSVII Pr. No αHHV Pr structures in the pdb have both a complete C-terminal tail and latch loop, so we used the alphafold3 server to predict the full interaction. We believe this reflects increased flexibility in the αHHV Pr latches. The

conserved Y and L residues from the MSA are highlighted. (c) The predicted hydrogen bond between the Q on the C-terminal tail and an R on the latch loop from the HSVII Pr AlphaFold structure.

	Fab5-HCMV Pr Class 1	Fab5-HCMV Pr Class 2	Fab5-HCMV Pr Class 3
**Data Collection and Processing**			
Magnification	EFTEM 165,000	EFTEM 165,000	EFTEM 165,000
Voltage (kV)	300	300	300
Electron exposure (e^-/Å^2)	50	50	50
Defocus range (μm)	-0.8-2.0	-0.8-2.0	-0.8-2.0
Pixel size (Å)	0.743	0.743	0.743
Symmetry imposed	C1	C1	C1
Initial particle number	2,795,045	2,795,045	2,795,045
Final particle images	429,127	538,690	487,966
Map resolution (Å)	2.72	2.57	2.69
FSC threshold	0.143	0.143	0.143
**Refinement**			
Initial model used	Ab initio	Ab initio	Ab initio
Model resolution (Å)	3.3	3.2	3.2
FSC threshold	0.5	0.5	0.5
Map sharpening B-factor	-117.0	-106.1	-114.5
Model Composition			
atoms	7863	7886	7019
Protein residues	586	582	482

Ligands	0	0	0
B factors (Å)			
Protein (min/max/mean)	92.34/263.7/139.98	76.96//251.89/131.40	96.89/301.92/129.36
R.M.S. Deviations			
Bond lengths (Å)	0.003	0.003	0.003
Bond angles (°)	0.516	1.510	0.515
Validation			
MolProbity score	1.12	1.49	1.36
Clashscore	1.54	2.82	2.29
Rotamer outliers (%)	0	1.68	0
Ramachandran plot			
Favored (%)	96.52	96.33	96.1
Allowed (%)	3.48	3.67	3.99
Outliers (%)	0	0	0
Model vs. Data			
CC (mask)	0.82	0.77	0.81
CC (box)	0.77	0.75	0.76
CC (peaks)	0.68	0.64	0.68
CC (volume)	0.82	0.77	0.81

Supplementary Table 1: Cryo-EM data collection, refinement, and validation statistics.
