# Supplementary material for: Antibody reveals conformational latch regulating dimerization in β- and γ-herpesvirus proteases
Source: Nat Commun. 2026 Jul 13;17:8590. doi: 10.1038/s41467-026-75438-2 (PMC13486704; doi:10.1038/s41467-026-75438-2)
Supplement: Supplementary file 2 [file 41467_2026_75438_MOESM2_ESM.pdf]

**The Description of Additional Supplementary Files**

**Supplementary Movie 1:** Atomic model of the Fab5-HCMV Pr complex morphing between classes 1, 2 and 3. HCMV Pr is observed twisting by 65° and tilting by 23° with respect to Fab5.

**Supplementary Movie 2**: Atomic model of HCMV Pr morphing between open and closed states of the latch loop (side chain atoms depicted as sticks). The movie begins with the latch loop in the closed position observed in HCMV Pr dimers (PDB: 1CMV) and then morphs into the open positions observed in classes 1, 2 and 3 of the Fab5-HCMV Pr complex. The C-terminal region of HCMV Pr is not shown.
