# Supplementary material for: Antibody reveals conformational latch regulating dimerization in β- and γ-herpesvirus proteases
Source: Nat Commun. 2026 Jul 13;17:8590. doi: 10.1038/s41467-026-75438-2 (PMC13486704; doi:10.1038/s41467-026-75438-2)
Supplement: Supplementary file 4 [file 41467_2026_75438_MOESM6_ESM.pdf]

nature portfolio

Peer Review File

Antibody Reveals Conformational Latch Regulating Dimerization in β- and γ-Herpesvirus Proteases

Corresponding Author: Professor Charles S Craik

This file contains all reviewer reports in order by version, followed by all author rebuttals in order by version.

Attachments originally included by the reviewers as part of their assessment can be found at the end of this file.

Version 0:

Reviewer comments:

Reviewer #1

(Remarks to the Author)
This manuscript reports a human Fab (Fab5) that inhibits HCMV protease (HCMV Pr) by binding a distal "latch loop", stabilizing a monomer-specific conformation that disfavors the disorder-to-order transition of the C terminus (α5/α6) and thereby inactivates the protease. Cryo EM maps define an epitope centered on residues 111–117 and motivate a model in which latch loop engagement perturbs dimerization and mispositions the oxyanion hole loop (OHL). Mutagenesis of latch loop/corresponding interface residues (e.g., E122, Y253, K255) ablates HCMV Pr activity; analogous mutations in KSHV Pr inactivate the enzyme, suggesting conservation across β/γ herpesviruses.

Major

-The text states the latch loop is "structurally conserved across all HHV Prs," and Fig. 4 is titled "conserved across the HHV family," yet functional tests are shown only for HCMV (β) and KSHV (γ); EBV is aligned but not functionally tested; α HVs (HSV 1/2, VZV) are not examined. I would suggest tempering claims to "β/γ herpesviruses" throughout and changing the title (e.g., "A latch loop mechanism controlling β/γ herpesvirus proteases revealed by an inhibitory antibody" as well as the heading of Fig. 4 accordingly. It would also help to add structure predictions of the alpha herpesvirus PR homologs and overlay of these with the beta- and gamma HV structures to illustrate the author's points.

-The authors report masses of Fab5 ~47 kDa; HCMV Pr ~32 kD,a and the proposed complex to be ~72 kDa (expected ~79 kDa). The S225M dimer is reported at 55 kDa (expected ~64 kDa). The explanation offered is co-elution. An alternative method, such as mass photometry, would help in convincing the reader.

-The structural mapping places the epitope on residues 111–117 ("latch loop"). Please test Fab5 binding to latch-mutant HCMV Pr (e.g., E122A, Y253A, K255A, and/or substitutions directly within 111–117). Loss (or weakening) of binding would solidify epitope assignment and the causal link between latch engagement, dimer disruption, and inhibition

-The Fab was built from an AlphaFold model and refined. The prediction scores should be reported (ipTM, PAE)

Minor
-The Discussion frames the latch site as a path to antivirals and cites intracellular antibody delivery, but the study is entirely biochemical; no cellular readouts are provided.
-Nine human herpesviruses (separate HHV 6A and HHV 6B) are currently recognized
-Add UL (HCMV) and ORF (KSHV) gene identifiers and construct lengths (domain boundaries) in Methods and in Fig. S1

Reviewer #2

(Remarks to the Author)
The manuscript entitled "Antibody Reveals Conformational Latch Controlling Herpesvirus Proteases" provides a

comprehensive biochemical and structural investigation of human cytomegalovirus protease (HCMV Pr) and its regulation by the latch loop. Through phage-display selection and cryo-EM analysis, the authors identified a Fab antibody (Fab5) that binds a distal latch-loop epitope and stabilizes the protease in its monomeric form. By combining enzymatic assays, mutagenesis, and structural comparisons, the study reveals how the latch loop controls the dimerization-dependent activation of HCMV Pr.

Overall, the data convincingly support the conclusion that Fab5 acts as a conformational probe to trap the monomeric state of HCMV Pr, thereby illuminating the dynamic role of the latch loop. However, the manuscript would benefit from clearer framing of its conceptual focus and a stronger articulation of how these findings advance current understanding beyond prior structural work on herpesvirus proteases.

Major Comments

1. The authors claim that SEC/MALS data demonstrate that Fab5 associates with monomeric HCMV Pr, but it remains unclear whether Fab5 selectively binds pre-existing monomers or actively shifts the monomer/dimer equilibrium. The evidence is weak by the SEC/MALS data of HCMV Pr S225M to make a such a conclusion.
2. The determination of a ~70 kDa Fab5-HCMV Pr complex at 2.6 Å resolution is an impressive methodological accomplishment, especially given the micromolar binding affinity. However, the manuscript's claim that this strategy can be readily extended to other herpesvirus proteases seems overly optimistic. The authors should therefore temper their statement and present this result as a proof-of-principle rather than a generally applicable approach.
3. The structural comparison and sequence alignment include HCMV Pr, KSHV Pr and EBV Pr (Fig. 4b), while the functional validation is limited to HCMV (β-HHV) and KSHV (γ-HHV). Given that EBV Pr is another representative of the γ-HHV subfamily, the absence of mutational and enzymatic characterization weakens the claim of conservation "across HHV subfamilies."
4. Previous structural studies on KSHV, EBV, and VZV proteases have described similar disorder-to-order transitions at the C-terminal tail during dimerization. The authors should highlight more clearly what is newly revealed here.

Minor Comments

Line 98, the strong phrasing (e.g., "This was the first evidence that Fab5 is an allosteric inhibitor") should be revised to a more cautious formulation.

Line 178-184 The authors have beautifully demonstrated the conservation of the latch mechanism in KSHV Pr (a γ-herpesvirus). However, as herpesviruses are divided into three subfamilies (α, β, γ), the claim of a universal mechanism would be significantly strengthened by addressing the α-herpesviruses. The article notes that the tri-residue motif is present in all β and γ members, but a sequence alignment for α-herpesviruses (e.g., HSV-1) and its functional validation are lacking. Given potential sequence and structural divergence, have the authors ruled out the possibility that α-herpesvirus proteases employ a structurally analogous, yet sequence-divergent, latch? Exploration of this subfamily, either through bioinformatic analysis or experiment, is crucial to support the broader implications of the findings. Furthermore, if the mechanism is not conserved in α-herpesviruses, it would be interesting to speculate whether its presence is uniquely important for the biology of β- and γ-herpesviruses, potentially even relating to the oncogenic potential of viruses like HCMV.

Line 108-110 While the data show that Fab5 sequesters monomers, it remains unclear if it simply binds free monomers or actively dissociates dimers. To clarify this, did you test whether incubation of Fab5 with pre-formed, purified HCMV Pr dimer leads to a reduction of the dimer peak in SEC analysis?

Reviewer #3

(Remarks to the Author)

Zimanyi et. al. investigate the structure and function of the human cytomegalovirus (HCMV) serine protease encoded by UL80. The protease is essential for viral capsid maturation, and is a druggable target for both HCMV and related herpesviruses. To investigate the protease, the authors first screened a phage display library for Fab fragments that bound the protease. One of those Fabs, termed Fab5, also disrupted the protease's catalytic activity. To determine the mechanism behind Fab5's disruption of the protease, the authors then solved a cryo-EM structure of the Fab5/UL80 protease complex. In the structure, Fab5 interacts with residues 111-117 of the protease, preventing the protease's C-terminal tail from forming a dimeric interface and assembling into the dimeric, active form of the protein. The authors validate the cryo-EM model by mutating residues involved in the latch loop/C-terminal tail interaction and find both that these mutants fail to dimerize and that they lose their catalytic activity. The authors also note that the overall structure of herpesvirus serine proteases are well-conserved between the human herpesviruses, and demonstrate that corresponding mutations to the KSHV serine protease completely abolish its catalytic activity.

Overall, the findings are exciting, with clear implications for structure-guided drug design. The paper is also well written, with data that support the authors' conclusions. However, one major critique is that not all of the cryo-EM map is at the reported resolution of ~2.7Å, and large portions of the density (most corresponding to parts of the viral protease away from the antibody binding site) are at too low a resolution to support accurate atomic model building. Obtaining a cryo-EM structure of an ~70kDa complex is impressive, but the limitations of the cryo-EM map and the corresponding atomic model need to be clearer.

Major comments:

1. Given the small size of the protein complex (~70kDa), obtaining a map with a resolution suitable for building an atomic model is an impressive feat. Both the antibody and the latch loop are clearly at a density appropriate to build an atomic model. However, while portions of the three cryo-EM maps in the paper have density consistent with the reported resolution of ~2.7Å, other regions are at substantially lower resolution. Because of the lower resolution (below 4Å) and the inability to trace the peptide backbone or accurately place side chains, I do not think that it is appropriate to build an atomic model into these low-density regions. Rigid-body docking of the available UL80 crystal structure into the density, truncating chains when the map drops below ~4Å resolution, and/or color-coding low-resolution density to track movements instead of building into it, would all give a more accurate representation of the data. It would also be appropriate to include a local resolution map to illustrate where these regions of lower resolution are.

2. In addition to regions with lower resolution, all three maps demonstrate a substantial amount of over-fitting, evidenced by noise/spikiness in the maps even at moderate contour levels. This may be the result of 3D refinement using a mask that was too tight.

3. In the analysis of enzyme kinetics in Figure S3c, fitting of the Lineweaver-Burke plot at 1.5µM of Fab5 is poor. Is there a reason why the datapoint with the highest substrate concentration was such an outlier?

Minor comments:
1. In figure S4, it is difficult to determine what loops belong to what protein or where the catalytic triad is in S4b and S4c. Because of the shading, in 4b and 4c, it is not clear that two protein structures are superimposed. In S4a, having a protein loop that is both green and orange is also difficult to interpret. Labels or a key within the figure (rather than just the legend) could also improve clarity.

Version 1:

Reviewer comments:

Reviewer #1

(Remarks to the Author)
I thank the authors for their thorough and thoughtful revisions, which have substantially improved the manuscript. The revised title, tempered conservation claims, and expanded structural analysis of α-HHV proteases address my original concerns well. The rebuttal was detailed and clearly written.
However, upon closer re-reading of the revised manuscript, several issues have come to my attention that I believe should be addressed before publication. These concern the enzymological interpretation, quantitative rigor of certain mechanistic claims, and some aspects of the cryo-EM structural analysis. I recognize that raising new points in a second round is not ideal, and I apologize for the additional burden. Nevertheless, I believe these issues are substantive enough that addressing them, primarily through textual revisions rather than new experiments, would meaningfully strengthen the manuscript's impact and accuracy.

Major Comments

1. The "non-competitive inhibition" classification is inconsistent with the proposed mechanism
The section heading (line 89) states "Fab5 is a Non-Competitive Inhibitor" and lines 100–102 describe the kinetic pattern as "consistent with a non-competitive mode of inhibition." However, the authors' own data in Fig. S3d show that Km increases from 50.5 ± 5.1 µM (no Fab5) to 59.36 ± 11.8 µM (1.5 µM Fab5), while Vmax decreases consistently from 0.00254 to 0.00144 µM/sec. By definition, a pure non-competitive inhibitor leaves Km unchanged and only reduces Vmax. If Km increases, the pattern is formally more consistent with mixed inhibition.
More fundamentally, the proposed mechanism — that Fab5 sequesters monomers and thereby depletes the pool of catalytically competent dimer (lines 117–121) — does not fit any classical steady-state inhibition model. In classical non-competitive inhibition, the inhibitor binds both free enzyme (E) and enzyme–substrate complex (ES) with equal affinity. Fab5 cannot bind the dimer (the active species) at all; it exclusively binds monomers. What the authors describe is an equilibrium perturbation mechanism, where Fab5 shifts the monomer–dimer equilibrium by removing monomers from the pool. The apparent decrease in Vmax therefore reflects reduced concentration of active enzyme, not classical inhibition of an enzyme–substrate interaction.
I suggest the authors either (a) re-classify the inhibition as "mixed" or "apparent non-competitive" with an explicit acknowledgement that the Lineweaver–Burk framework is an approximation given the monomer-depletion mechanism, or (b) reframe the inhibition entirely in terms of the equilibrium shift model, which is more mechanistically accurate.

2. Assay conditions and their implications for the equilibrium shift argument
The steady-state kinetics assays are performed at 500 nM HCMV Pr in 10% glycerol (lines 322–324), while the IC50 assays use 500 nM HCMV Pr in 5% glycerol (line 347). The dimerization Kd is reported as ~70 µM in saline and ~5.8 µM in 10% glycerol (lines 115–116). Even under the most favorable glycerol-stabilizing conditions, 500 nM is approximately 10-fold below the dimer Kd, meaning that the enzyme is already predominantly monomeric and thus largely inactive in both assays. The small fraction of dimer present at equilibrium is what provides the observed activity.
This has two important implications. First, the manuscript's comparison of Fab5 Kd (1.6 µM) to a "44-fold lower" dimerization Kd (Fig. 4d legend, line 680) uses the saline Kd of 70 µM. However, the steady-state kinetics assays are performed in 10% glycerol buffer where the dimer Kd is ~5.8 µM. Under these conditions the affinity advantage is only ~3.6-fold, substantially

weakening the thermodynamic argument. Second, the IC50 assay uses 5% glycerol, where the dimer Kd is presumably intermediate between 5.8 and 70 µM but is not reported. The manuscript should explicitly reconcile which dimerization Kd applies under which assay conditions and present the equilibrium shift argument using condition-matched values.
I recommend a brief discussion acknowledging that at the assay concentration of 500 nM, the enzyme is predominantly monomeric under all tested buffer conditions, and clarify the buffer-dependent dimerization Kd values used in the thermodynamic comparison. The 44-fold claim in Fig. 4d should either use the condition-matched Kd or explicitly note that it refers to saline conditions. This is a textual clarification, not a request for new experiments.

3. Causality of the latch–dimerization relationship requires more cautious framing
The data convincingly show that latch motif mutations (E122A, Y253A, K255A) abolish both activity and dimerization. However, this establishes correlation, not directionality. It is not demonstrated whether latch engagement drives the disorder-to-order transition of the C-terminus and thereby initiates dimerization, or whether dimerization occurs first and the latch locks the C-terminus in place afterwards. No structural intermediate of a pre-dimer, latch-closed monomer has been observed.
The Discussion appropriately refers to a "bidirectional relationship" (line 220). However, the Results section uses directional and causal language. The section heading "The Latch Loop Governs HCMV Pr Dimerization and Activity" (line 159) implies directionality. Lines 137–138 state that "disrupting the interaction between the C-terminal tail and the latch loop prevents the entire C-terminal region from folding," and lines 155–157 state "Disrupting the latch-tail interface prevents the C-terminal region from adopting its ordered form, revealing a previously unknown mechanism." This causal framing in the Results is not consistent with the appropriately hedged bidirectional model in the Discussion.
I suggest moderating the causal language in the Results to match the Discussion's bidirectional framing.

4. Interpretation of absent α5/α6 density and the OHL displacement
The authors state that "no EM density corresponding to [α5 and α6] helices appears even at low thresholds" (lines 136–137) and write that "This suggests that disrupting the interaction between the C-terminal tail and the latch loop prevents the entire C-terminal region from folding" (lines 137–139). I appreciate the use of "suggests" here. However, it is well established that the HCMV Pr monomer has a disordered C-terminus. Since the Fab5 complex is monomeric by definition, the absence of α5/α6 density is expected for any monomeric species and does not independently demonstrate that latch disruption is the cause of C-terminal disorder. The observation is consistent with the model but the later passage at lines 155–157, which states that "Disrupting the latch-tail interface prevents the C-terminal region from adopting its ordered form, revealing a previously unknown mechanism," reverts to causal language that is stronger than the underlying data support.
Regarding the OHL, the authors appropriately note that they "can infer the orientation of the OHL from low-resolution density" (line 143) and that the OHL "appears to open by 5.6Å" (line 144). I appreciate the hedging language. However, the local resolution map in Fig. S6B indicates that the active site region falls in the 4–5 Å range. A precise measurement of 5.6 Å from a region at this resolution may convey more confidence than warranted. Reporting this as "approximately 5–6 Å" or "density consistent with an open OHL conformation" would better reflect the underlying data quality.
I recommend reframing lines 155–157 to be consistent with the "suggests" language used at lines 137–139. Consider softening the OHL displacement to an approximate range rather than a precise value.

5. K255A thermal destabilization and DSF concentration discrepancy
The DSF data (Fig. 3d) show that K255A exhibits a Tm of 44.85 ± 0.09°C versus 48.28 ± 0.05°C for wild type, a decrease of 3.4°C. The figure legend (line 670), however, states the decrease is "4°C." This rounding discrepancy should be corrected for consistency.
More substantively, there is a discrepancy in the reported DSF protein concentration: the Methods section states "Protein (2 µM)" (line 394), whereas the Results state that experiments were "performed at an HCMV Pr concentration of 4µM" (line 176). This should be corrected.
Regarding interpretation, a 3.4°C Tm decrease is not negligible and raises the question of whether K255A's loss of activity is partly attributable to local destabilization of the C-terminal region rather than exclusively to disruption of the latch–tail interaction. The authors state that these results "indicate that the latch mutations do not substantially compromise the overall protein fold or stability" (lines 180–181), but the K255A Tm shift is notably larger than the other mutants (≤1°C) and deserves individual comment. Additionally, DSF at the reported concentrations (2 or 4 µM) is well below the dimerization Kd, meaning it primarily measures monomer thermal stability and does not address whether K255A specifically destabilizes the dimer interface.
I recommend correcting the concentration discrepancy between Methods and Results, correct the rounding in the figure legend, and add a sentence noting the K255A Tm shift as a caveat. Acknowledge that DSF at sub-Kd concentrations primarily reports on monomer stability.

Reviewer #2

(Remarks to the Author)
I thank the authors for their careful and thoughtful responses to my previous comments. The revised manuscript has been substantially improved in clarity, scope, and conceptual framing. The authors have appropriately tempered several claims, strengthened the discussion of mechanism, and clarified the limits of their conclusions.
Regarding the Fab5 binding mechanism, the expanded thermodynamic explanation of the monomer–dimer equilibrium and the inclusion of affinity comparisons (Fab5–HCMV Pr vs. HCMV Pr dimerization) significantly strengthen the argument. I also appreciate the more cautious positioning of the cryo-EM strategy as a proof-of-principle rather than a universally applicable method. The revisions to the Discussion appropriately reflect the case-dependent nature of extending this approach to other herpesvirus proteases.

Importantly, the authors have more clearly articulated what is conceptually novel relative to prior structural studies on herpesvirus proteases, namely the identification of the latch loop as a regulatory element that stabilizes the C-terminal disorder-to-order transition during dimerization, and the use of an antibody as a conformational probe to trap the monomeric state. This framing now makes the advance over previous work clear.

Reviewer #3

(Remarks to the Author)
Zimanyi et. al. have responded with an updated version of their manuscript. The conclusions remain well-founded and the story interesting; however, I still have concerns about how the authors have addressed model building into low-density regions of the cryo-EM map.

Major comments:
1. The authors have clarified their model-building approach, but the issue of building a model into low-density regions of the cryo-EM map remains. There is not enough density in any of the three maps to build the distal parts of the protease, and the parts of the atomic model that include these regions are not supported by the cryo-EM data. Docking the existing crystal structure (as an unmodified atomic model) into the cryo-EM density, to show how the protease is oriented and where the loops likely are, is perfectly acceptable. Using the crystal structure as a starting point in model refinement into the cryo-EM density is also widely accepted. The problem is that the newly generated models in this manuscript were not truncated where the cryo-EM density is insufficient to accurately place residues. They will not have a structure identical to the one defined by x-ray crystallography, and being unable to see the distal parts of the protease at 4Å or better resolution means it is not possible to accurately place those regions of the protein in an atomic model.

If the depiction of the map in Fig. S6 is misleading and sufficient cryo-EM density exists to build the models in Fig. S6 and Table S1 are present, I would request to see the PDB/EMDB validation report and the Phenix refinement map/model correlation coefficient graph for the refined map/model combinations. If sufficient density is not present to support residue placement, the newly generated atomic models should be truncated to include only residues the cryo-EM density can support.

Version 2:

Reviewer comments:

Reviewer #1

(Remarks to the Author)
I thank the authors for their careful and thorough responses to my comments. The revisions, including the reframing of the inhibition mechanism, the condition-matched thermodynamic comparisons, the moderated causal language in the Results, and the revised interpretation of the cryo-EM density, address all of my concerns. I have no further comments and support the publication of the manuscript.

Reviewer #3

(Remarks to the Author)
The authors have addressed my concerns for the manuscript in their most recent response to reviewer comments.

Open Access This Peer Review File is licensed under a Creative Commons Attribution 4.0 International License, which permits use, sharing, adaptation, distribution and reproduction in any medium or format, as long as you give appropriate credit to the original author(s) and the source, provide a link to the Creative Commons license, and indicate if changes were made.
In cases where reviewers are anonymous, credit should be given to 'Anonymous Referee' and the source.
The images or other third party material in this Peer Review File are included in the article's Creative Commons license, unless indicated otherwise in a credit line to the material. If material is not included in the article's Creative Commons license and your intended use is not permitted by statutory regulation or exceeds the permitted use, you will need to obtain permission directly from the copyright holder.
To view a copy of this license, visit https://creativecommons.org/licenses/by/4.0/

REVIEWER COMMENTS

Reviewer #1 (Remarks to the Author):

This manuscript reports a human Fab (Fab5) that inhibits HCMV protease (HCMV Pr) by binding a distal "latch loop", stabilizing a monomer-specific conformation that disfavors the disorder-to-order transition of the C terminus (α5/α6) and thereby inactivates the protease. Cryo EM maps define an epitope centered on residues 111–117 and motivate a model in which latch loop engagement perturbs dimerization and mispositions the oxyanion hole loop (OHL). Mutagenesis of latch loop/corresponding interface residues (e.g., E122, Y253, K255) ablates HCMV Pr activity; analogous mutations in KSHV Pr inactivate the enzyme, suggesting conservation across β/γ herpesviruses.

Major

1. The text states the latch loop is "structurally conserved across all HHV Prs," and Fig. 4 is titled "conserved across the HHV family," yet functional tests are shown only for HCMV (β) and KSHV (γ); EBV is aligned but not functionally tested; α HVs (HSV 1/2, VZV) are not examined. I would suggest tempering claims to "β/γ herpesviruses" throughout and changing the title (e.g., "A latch loop mechanism controlling β/γ herpesvirus proteases revealed by an inhibitory antibody" as well as the heading of Fig. 4 accordingly. It would also help to add structure predictions of the alpha herpesvirus PR homologs and overlay of these with the beta- and gamma HV structures to illustrate the author's points.

Response to reviewer:
We thank the reviewer for this point and agree that claims of pan-HHV conservation should be appropriately scoped to reflect the data. We made the following changes:

1. *Title is now "Antibody Reveals Conformational Latch Regulating Dimerization in β- and γ-Herpesvirus Proteases"*
2. *Abstract: We clarified that "This loop is structurally conserved across all HHV Prs, with a critical 3-residue motif present in β- and γ-herpesvirus proteases. We demonstrate the functional importance of this motif in both HCMV Pr and Kaposi's Sarcoma-associated herpesvirus (KSHV) Pr."*
3. *Introduction: We specify that the three-residue motif is present in five of nine HHV family members spanning β- and γ-herpesvirus proteases.*
4. *Results: We revised a section title to "Structural and Functional Conservation of the Latch Loop in β- and γ-herpesvirus proteases" and added discussion of the α-HHVs. We also performed a sequence alignment across all HHV Pr sequences and made a new supplemental figure with this alignment and views of the α-HHV Pr latch. Our comparative structural analysis shows that while the latch loop is structurally present in α-HHVs, the interface involves more subtle interactions with the C-terminal tail than those observed in β- and γ-HHVs.*

5. *Discussion: We have added more explicit acknowledgement of our study's scope: "While our functional studies are limited to HCMV and KSHV, the sequence and structural conservation of the three-residue latch motif across β- and γ-HHV Prs strongly suggests this regulatory mechanism extends to EBV Pr and other members of these subfamilies." We also revised multiple other statements to temper claims that universally applied to HHV Prs.*

2. The authors report masses of Fab5 ~47 kDa; HCMV Pr ~32 kD,a and the proposed complex to be ~72 kDa (expected ~79 kDa). The S225M dimer is reported at 55 kDa (expected ~64 kDa). The explanation offered is co-elution. An alternative method, such as mass photometry, would help in convincing the reader.

Response to reviewer:
We have streamlined the results section to focus on the key conclusion that the SEC/MALS demonstrates a 1:1 stoichiometry between Fab5 and monomeric HCMV Pr. The observed molecular weights are fully consistent with this stoichiometry and exclude the possibility of Fab5 binding to dimers, which would yield substantially larger complexes (108-159 kDa, depending on 1 or 2 Fabs bound to a dimer).

Furthermore, our cryo-EM data provide direct validation that Fab5 forms a 1:1 complex with HCMV Pr. When Fab5 binds the latch loop, it sterically hinders dimer formation. We agree with the reviewer that mass photometry would help convince the reader of the SEC/MALS results alone, however we believe that our cryo-EM data provides compelling evidence that for the 1:1 stoichiometry of the Fab5-HCMV Pr complex.

3. The structural mapping places the epitope on residues 111–117 ("latch loop"). Please test Fab5 binding to latch-mutant HCMV Pr (e.g., E122A, Y253A, K255A, and/or substitutions directly within 111–117). Loss (or weakening) of binding would solidify epitope assignment and the causal link between latch engagement, dimer disruption, and inhibition.

Response to reviewer:
We appreciate the suggestion to further validate our epitope and our proposed mechanism. Given that the Fab5-HCMV Pr interface is well resolved with high model confidence, there is no alternative to the epitope we describe. We believe our existing data is definitive that Fab5 binds the latch loop, though we acknowledge that binding studies with the mutants would offer additional support.

Our chain of reasoning to propose the mechanism is as follows: Fab5 binds the latch loop (cryo-EM structures), this binding is only possible when the latch is open, Fab5 binding shifts HCMV Pr towards its monomeric state and inhibits activity, and latch mutations also eliminate dimerization and activity. Together, we believe these data demonstrate the causal link between latch engagement and dimerization.

4. The Fab was built from an AlphaFold model and refined. The prediction scores should be reported (ipTM, PAE)

Response to reviewer:
We have added the AlphaFold prediction scores of the Fab5 starting model to supplementary figure S6.

Minor
1. The Discussion frames the latch site as a path to antivirals and cites intracellular antibody delivery, but the study is entirely biochemical; no cellular readouts are provided.

Response to reviewer:
Our goal was to frame all statements about therapeutic potential as speculation and future directions. We made many updates to our discussion section to address the manuscript's clarity and scope and have taken additional steps to strike a speculative tone. The primary contribution of this work is the identification of an allosteric site that could be exploited in future therapeutic development efforts. We now explicitly present Fab5 as a proof-of-concept tool compound that validates the latch mechanism biochemically.

2. Nine human herpesviruses (separate HHV 6A and HHV 6B) are currently recognized

Response to reviewer:
We thank the reviewer for pointing this out and have changed the main text and figures to reflect this

3. Add UL (HCMV) and ORF (KSHV) gene identifiers and construct lengths (domain boundaries) in Methods and in Fig. S1

Response to reviewer:
We have added gene identifiers for UL80 and ORF17 to the methods section. Figure S1 includes a schematic of the UL80 gene as well.

Reviewer #2 (Remarks to the Author):

The manuscript entitled "Antibody Reveals Conformational Latch Controlling Herpesvirus Proteases" provides a comprehensive biochemical and structural investigation of human cytomegalovirus protease (HCMV Pr) and its regulation by the latch loop. Through phage-display selection and cryo-EM analysis, the authors identified a Fab antibody (Fab5) that binds a distal latch-loop epitope and stabilizes the protease in its monomeric form. By combining enzymatic assays, mutagenesis, and structural comparisons, the study reveals how the latch loop controls the dimerization-dependent activation of HCMV Pr.
Overall, the data convincingly support the conclusion that Fab5 acts as a conformational probe to trap the monomeric state of HCMV Pr, thereby illuminating the dynamic role of the latch loop. However, the manuscript would benefit from clearer framing of its conceptual focus and a stronger articulation of how these findings advance current understanding beyond prior structural work on herpesvirus proteases.

Major Comments

1. The authors claim that SEC/MALS data demonstrate that Fab5 associates with monomeric HCMV Pr, but it remains unclear whether Fab5 selectively binds pre-existing monomers or actively shifts the monomer/dimer equilibrium. The evidence is weak by the SEC/MALS data of HCMV Pr S225M to make a such a conclusion.

Response to reviewer:
We appreciate this important point regarding the mechanism of Fab5 binding. We believe that the presented scenarios where Fab5 binds pre-existing monomers or actively shifts the monomer/dimer equilibrium are not mutually exclusive. Fab5 binds monomeric HCMV Pr, and because Fab5/HCMV Pr complex formation is thermodynamically favorable to HCMV Pr dimerization, the equilibria are shifted towards both the monomeric state as well as Fab5-HCMV Pr complexes.

Our SEC/MALS and cryoEM data consistently demonstrate that Fab5 engages monomeric HCMV Pr, and we observe no evidence that it can bind dimers. The Kd of Fab5 for HCMV Pr is 1.6 μM, which is substantially lower than the Kd for HCMV Pr dimerization, which is 70 μM[1]. This ~44-fold difference in affinity favors the Fab5-monomer complex and thereby shifts the equilibrium. If we consider the equilibrium scheme of HCMV Pr dimer $\rightleftharpoons$ HCMV Pr monomer $\rightleftharpoons$ Fab5/HCMV Pr complex, as Fab5 binds monomers, it depletes the monomer pool, reestablishing the equilibrium to greatly favor the monomeric state and Fab5-HCMV Pr complexes. We have included these points into the results section along with the SEC/MALS data because this thermodynamic concept explains the 1:1 stoichiometry. This all extends to HCMV Pr S225M as well.

2. The determination of a ~70 kDa Fab5-HCMV Pr complex at 2.6 Å resolution is an impressive methodological accomplishment, especially given the micromolar binding affinity. However, the manuscript's claim that this strategy can be readily extended to other herpesvirus proteases seems overly optimistic. The authors should therefore temper their statement and present this result as a proof-of-principle rather than a generally applicable approach.

Response to reviewer: We appreciate the reviewer highlighting this point. We agree that while our work demonstrates the feasibility of cryoEM for Fab epitope determination, extension to other herpesvirus proteases will be case-dependent. We revised the discussion to reflect the accomplishment and its limitations.

Indeed, at the early stage of this project, the goal of determining high resolution structures of Fabs bound to small, flexible proteins seems challenging. However, after the success of the Fab5-HCMV Pr structure, we have now solved multiple cryoEM structures of Fabs, including a Fab bound to a 15 amino acid peptide. We thus believe that Fab epitope mapping via cryoEM could be extended to other herpesvirus protease binders or other Fab-complexes.

3. The structural comparison and sequence alignment include HCMV Pr, KSHV Pr and EBV Pr (Fig. 4b), while the functional validation is limited to HCMV (β-HHV) and KSHV (γ-HHV). Given that EBV Pr is another representative of the γ-HHV subfamily, the absence of mutational and enzymatic characterization weakens the claim of conservation "across HHV subfamilies."

Response to reviewer:

We agree with the reviewer that our claims should match the scope of our functional validation. We have been more careful about claiming that certain concepts may apply to all HHV Prs. We more explicitly acknowledge that our functional data studies are limited to HCMV and KSHV. However, we note that conservation of the three-residue motif across all β- and γ-HHV Prs strongly suggest this regulatory mechanism extends throughout these two subfamilies. The motif forms prominent salt-bridge and hydrophobic interactions that are structurally present in EBV Pr as well. We also add structural analysis of α-HHV Prs to the results and Fig. 4 to illustrate differences in latch-C-terminus interactions.

We would like to highlight the following changes:

1. *Title is now "Antibody Reveals Conformational Latch Regulating Dimerization in β- and γ-Herpesvirus Proteases"*
2. *Abstract: We clarified that "This loop is structurally conserved across all HHV Prs, with a critical 3-residue motif present in β- and γ-herpesvirus proteases. We demonstrate the functional importance of this motif in both HCMV Pr and Kaposi's Sarcoma-associated herpesvirus (KSHV) Pr."*
3. *Introduction: We specify that the three-residue motif is present in five of nine HHV family members spanning β- and γ-herpesvirus proteases.*
4. *Results: We revised a section title to "Structural and Functional Conservation of the Latch Loop in β- and γ-herpesvirus proteases" and added discussion of the α-HHVs. We also performed a sequence alignment across all HHV Pr sequences and made a new supplemental figure with this alignment and views of the α-HHV Pr latch. Our comparative structural analysis shows that while the latch loop is structurally present in α-HHVs, the interface involves more subtle interactions with the C-terminal tail than those observed in β- and γ-HHVs.*
5. *Discussion: We have added more explicit acknowledgement of our study's scope: "While our functional studies are limited to HCMV and KSHV, the sequence and structural conservation of the three-residue latch motif across β- and γ-HHV Prs strongly suggests this regulatory mechanism extends to EBV Pr and other members of these subfamilies." We also revised multiple other statements to temper claims that universally applied to HHV Prs.*

4. Previous structural studies on KSHV, EBV, and VZV proteases have described similar disorder-to-order transitions at the C-terminal tail during dimerization. The authors should highlight more clearly what is newly revealed here.

Response to reviewer:
We made changes to more explicitly state our novel contribution in context of previous work. Previous studies indeed established that a disorder-to-order transition occurs and demonstrated how important the C-terminus is for HHV Pr activity, however the molecular mechanisms governing this transition remained unclear. Our work reveals the latch loop as a critical structural element that secures this transition once it is complete.

We made the following changes:

1. *Introduction: Transition between paragraphs stating that the molecular mechanism of the disorder-to-order transition has remained unclear, and that inhibitors are great tools for studying molecular mechanisms.*
2. *Results: At the end of the cryo-EM section, we add a summary of the structural significance of our findings and how they contribute to understanding HHV Pr proteolysis.*

Minor Comments

Line 98, the strong phrasing (e.g., "This was the first evidence that Fab5 is an allosteric inhibitor") should be revised to a more cautious formulation.

Response to reviewer:
Changed to "This indicated that Fab5 may be an allosteric inhibitor."

Line 178-184 The authors have beautifully demonstrated the conservation of the latch mechanism in KSHV Pr (a γ-herpesvirus). However, as herpesviruses are divided into three subfamilies (α, β, γ), the claim of a universal mechanism would be significantly strengthened by addressing the α-herpesviruses. The article notes that the tri-residue motif is present in all β and γ members, but a sequence alignment for α-herpesviruses (e.g., HSV-1) and its functional validation are lacking. Given potential sequence and structural divergence, have the authors ruled out the possibility that α-herpesvirus proteases employ a structurally analogous, yet sequence-divergent, latch? Exploration of this subfamily, either through bioinformatic analysis or experiment, is crucial to support the broader implications of the findings. Furthermore, if the mechanism is not conserved in α-herpesviruses, it would be interesting to speculate whether its presence is uniquely important for the biology of β- and γ-herpesviruses, potentially even relating to the oncogenic potential of viruses like HCMV.

Response to reviewer:
We appreciate the reviewer's comment as well as the suggestion to more thoroughly address the α-HHV Prs. To expand on our response to major comment 3, we have added a supplementary figure that aligns HHV Pr structures and sequences across the three subfamilies.

1. *We included a new supplementary figure (Fig. S8) with a multiple sequence alighment across all known human herpesvirus protease sequences and highlighting what we believe to be the critical residue interactions between the α-HHV Pr latch and C-terminus. This analysis shows that while the latch is present, the interactions are more subtle and lack the salt bridges and large hydrophobic contacts that define the interface in β- and γ-HHV Prs.*
2. *Results: We discuss the possibility of a structurally analogous mechanism. The α-HHV Prs may use an analogous but sequence-divergent latch mechanism, however the absence of the conserved motif and the reduced contact interface between the latch and the tail indicate that it may play a less prominent regulatory role than in β- and γ-HHV Prs.*

3. *We revised claims in the manuscript overall to specify that our functional validation applies to the β- and γ-HHV Prs and tempered claims of conservation across the whole family to state only what we can support with our data. We revised the title as well.*

Line 108-110 While the data show that Fab5 sequesters monomers, it remains unclear if it simply binds free monomers or actively dissociates dimers. To clarify this, did you test whether incubation of Fab5 with pre-formed, purified HCMV Pr dimer leads to a reduction of the dimer peak in SEC analysis?

Response to reviewer:
This is an interesting mechanistic question, and this is related to our response to comment #1. We believe our data strongly support a model where Fab5 binds HCMV Pr monomers rather than binding dimers and causing them to dissociate. Fab5 shifts the HCMV Pr monomer-dimer equilibrium heavily towards monomers and Fab5-HCMV Pr complexes.

Our other data support this as well. Our structures show that Fab5 is sterically occluded from binding the latch loop in the dimeric configuration, suggesting that it binds monomers. Also, we see no evidence of Fab5-dimer binding in either our biochemical or structural characterization. We took another close look at our cryoEM particle classification steps and do not find Fab5-dimer complexes.

We have added this mechanistic discussion of the equilibria and how Fab5 drives HCMV Pr towards monomeric states at the end of the first results section.

Reviewer #3 (Remarks to the Author):

Zimanyi et. al. investigate the structure and function of the human cytomegalovirus (HCMV) serine protease encoded by UL80. The protease is essential for viral capsid maturation, and is a druggable target for both HCMV and related herpesviruses. To investigate the protease, the authors first screened a phage display library for Fab fragments that bound the protease. One of those Fabs, termed Fab5, also disrupted the protease's catalytic activity. To determine the mechanism behind Fab5's disruption of the protease, the authors then solved a cryo-EM structure of the Fab5/UL80 protease complex. In the structure, Fab5 interacts with residues 111-117 of the protease, preventing the protease's C-terminal tail from forming a dimeric interface and assembling into the dimeric, active form of the protein. The authors validate the cryo-EM model by mutating residues involved in the latch loop/C-terminal tail interaction and find both that these mutants fail to dimerize and that they lose their catalytic activity. The authors also note that the overall structure of herpesvirus serine proteases are well-conserved between the human herpesviruses, and demonstrate that corresponding mutations to the KSHV serine protease completely abolish its catalytic activity.

Overall, the findings are exciting, with clear implications for structure-guided drug design. The paper is also well written, with data that support the authors' conclusions. However, one major critique is that not all of the cryo-EM map is at the reported resolution of ~2.7Å, and large portions of the density (most corresponding to parts of the viral protease away from the antibody binding site) are at too low a resolution to support accurate atomic model building. Obtaining a cryo-EM

structure of an ~70kDa complex is impressive, but the limitations of the cryo-EM map and the corresponding atomic model need to be clearer.

Major comments:
1. Given the small size of the protein complex (~70kDa), obtaining a map with a resolution suitable for building an atomic model is an impressive feat. Both the antibody and the latch loop are clearly at a density appropriate to build an atomic model. However, while portions of the three cryo-EM maps in the paper have density consistent with the reported resolution of ~2.7Å, other regions are at substantially lower resolution. Because of the lower resolution (below 4Å) and the inability to trace the peptide backbone or accurately place side chains, I do not think that it is appropriate to build an atomic model into these low-density regions. Rigid-body docking of the available UL80 crystal structure into the density, truncating chains when the map drops below ~4Å resolution, and/or color-coding low-resolution density to track movements instead of building into it, would all give a more accurate representation of the data. It would also be appropriate to include a local resolution map to illustrate where these regions of lower resolution are.

Response to reviewer:
We appreciate the reviewer's positive assessment of our work and for the constructive feedback. Indeed, the reported resolution is the overall resolution. We included a supplementary figure of our map colored by local resolution. The goal of our structural study is to determine how the Fab binds the protease. We reasoned that we would be able to resolve the Fab-epitope interface even in other parts of the Fab (such as the constant domain) or the target protein (distal regions) would not be well resolved due to conformational flexibility. This is indeed what we have accomplished. By now, we have extended this approach to several different Fabs, including one bound to a short peptide, and achieved sub-3Å resolution at the interface allowing for accurate model building. These manuscripts are still in preparation.

Our modeling strategy was in fact quite similar to what the reviewer recommends. We first generated all flexible loops of HCMV Pr as peptides in Coot and systematically evaluated their fit into density that did correspond to Fab5. The latch loop peptide showed the clearest fit, which prompted us to build it de novo as far as the high-resolution density would allow (residues 111-117 and about 30 residues, including flanking helices). We then rigid-body docked the HCMV Pr crystal structure (pdb: 1CMV) and appended it to our model. The helices flanking the latch loop provided landmarks for orienting the structure, and distal densities aligned well with the rigid-body fit. Regions where density quality was insufficient for confident modelling were retained from the crystal structure with minimal adjustment if they were in the protein core.

We made the following changes: In our methods section, we clarified our modelling approach to explicitly describe how the model was built. We added local resolution maps to Fig. S6 at low and high thresholds. We note that local resolution tools in relion, phenix and cryosparc report optimistic resolution values that do not seem to capture the full visual variation of density across the map. Among the three, cryosparc seemed to be the best fit. Nevertheless, these maps clearly show that the Fab5-latch loop interface exhibits the highest resolution, with decreasing resolution farther from the binding site. We also made changes in the cryo-EM results section to clearly acknowledge the variable resolution across the map and note that our modelling is most confident at the interface.

2. In addition to regions with lower resolution, all three maps demonstrate a substantial amount of over-fitting, evidenced by noise/spikiness in the maps even at moderate contour levels. This may be the result of 3D refinement using a mask that was too tight.

Response to reviewer:
We thank the reviewer for the careful check of the maps. However, it is not clear to us that there is "substantial overfitting." We carefully re-examined our unsharpened maps and did not find signs of over-refining. Following the suggestion, we re-processed our data using multiple looser masks and obtained nearly identical results. It is possible that there is slight over-sharpening of the map.

3. In the analysis of enzyme kinetics in Figure S3c, fitting of the Lineweaver-Burke plot at 1.5µM of Fab5 is poor. Is there a reason why the datapoint with the highest substrate concentration was such an outlier?

Response to reviewer:
This last point is likely an outlier because substrate concentration and thus enzyme activity are near zero at that point, and subtle changes can cause large variations in a double-reciprocal plot. We considered excluding this point as a statistical outlier, particularly because the remaining points show excellent linear correlation. However, we kept the point for the sake of transparency. We have made a change where the point is graphed on the Lineweaver-Burk plot but is grayed out, and the remaining points are fit to the curve. This is noted in the figure description as well.

Minor comments:
1. In figure S4, it is difficult to determine what loops belong to what protein or where the catalytic triad is in S4b and S4c. Because of the shading, in 4b and 4c, it is not clear that two protein structures are superimposed. In S4a, having a protein loop that is both green and orange is also difficult to interpret. Labels or a key within the figure (rather than just the legend) could also improve clarity.

Response to reviewer:
We thank the reviewer for their constructive feedback and suggestions. We have changed Fig. S4 by adding the whole HCMV Pr structure and labelling the zone of interest to orient the reader. We have also made each model a single color and added labels to improve clarity.

Citations:

(1) Batra, R.; Khayat, R.; Tong, L. Molecular Mechanism for Dimerization to Regulate the Catalytic Activity of Human Cytomegalovirus Protease. *Nat. Struct. Biol.* **2001**, *8* (9), 810–817. https://doi.org/10.1038/nsb0901-810.

REVIEWER COMMENTS

Reviewer #1 (Remarks to the Author):

I thank the authors for their thorough and thoughtful revisions, which have substantially improved the manuscript. The revised title, tempered conservation claims, and expanded structural analysis of α-HHV proteases address my original concerns well. The rebuttal was detailed and clearly written.
However, upon closer re-reading of the revised manuscript, several issues have come to my attention that I believe should be addressed before publication. These concern the enzymological interpretation, quantitative rigor of certain mechanistic claims, and some aspects of the cryo-EM structural analysis. I recognize that raising new points in a second round is not ideal, and I apologize for the additional burden. Nevertheless, I believe these issues are substantive enough that addressing them, primarily through textual revisions rather than new experiments, would meaningfully strengthen the manuscript's impact and accuracy.

Response: We thank the reviewer for their careful analysis and constructive comments. These are all interesting discussion topics, and we agree that addressing them strengthens the mechanistic accuracy of the manuscript. Please find our responses below where the reviewer comments are italicized and our responses are not italicized.

Major Comments

1. The "non-competitive inhibition" classification is inconsistent with the proposed mechanism
The section heading (line 89) states "Fab5 is a Non-Competitive Inhibitor" and lines 100–102 describe the kinetic pattern as "consistent with a non-competitive mode of inhibition." However, the authors' own data in Fig. S3d show that Km increases from 50.5 ± 5.1 μM (no Fab5) to 59.36 ± 11.8 μM (1.5 μM Fab5), while Vmax decreases consistently from 0.00254 to 0.00144 μM/sec. By definition, a pure non-competitive inhibitor leaves Km unchanged and only reduces Vmax. If Km increases, the pattern is formally more consistent with mixed inhibition.
More fundamentally, the proposed mechanism — that Fab5 sequesters monomers and thereby depletes the pool of catalytically competent dimer (lines 117–121) — does not fit any classical steady-state inhibition model. In classical non-competitive inhibition, the inhibitor binds both free enzyme (E) and enzyme–substrate complex (ES) with equal affinity. Fab5 cannot bind the dimer (the active species) at all; it exclusively binds monomers. What the authors describe is an equilibrium perturbation mechanism, where Fab5 shifts the monomer–dimer equilibrium by removing monomers from the pool. The apparent decrease in Vmax therefore reflects reduced concentration of active enzyme, not classical inhibition of an enzyme–substrate interaction.
I suggest the authors either (a) re-classify the inhibition as "mixed" or "apparent non-competitive" with an explicit acknowledgement that the Lineweaver–Burk framework is an

approximation given the monomer-depletion mechanism, or (b) reframe the inhibition entirely in terms of the equilibrium shift model, which is more mechanistically accurate.

Response to comment 1: We thank the reviewer for this careful analysis and agree that our classification warrants revision. We agree that a modest Km increase alongside a consistent Vmax decrease is more consistent with mixed inhibition than non-competitive inhibition. This observation is consistent with prior work from our group showing that dimer-disrupting small molecules targeting HCMV Pr also produce mixed inhibition kinetics, suggesting that this kinetic profile is characteristic of compounds that disrupt the allosteric relationship between dimerization and ordering of the active site[1]. We agree with the reviewer that applying a classical steady-state definition to the relationship between Fab5 and HCMV Pr does not fully describe the mechanism of inhibition, so we reframed the inhibition in terms of the equilibrium shift model.

We made the following changes:

1) First results section header on line 89 revised from "Fab5 is a Non-Competitive Inhibitor that Isolates HCMV Pr Monomers" to "Fab5 Allosterically Inhibits HCMV Pr by Shifting the Monomer-Dimer Equilibrium"
2) The Fig. 1c legend was simplified to "Steady-state kinetics of HCMV Pr performed with increasing concentrations of Fab5."
3) In the results on line 100, we say "…as Fab5 concentration increases, the V_{max} of the reaction decreases and the K_m increases modestly, indicating that Fab5 does not compete with substrate at the active site. This kinetic profile is consistent with mixed-type kinetics previously observed for dimer-disrupting compounds targeting HCMV Pr, suggesting that Fab5 may act allosterically by disrupting dimerization[1]." This observation was key to establishing our hypothesis that Fab5 may disrupt dimerization and thus be an allosteric inhibitor, which led us to test the hypothesis by SEC/MALS. We conclude the section by framing the inhibition in terms of the equilibrium shift model.

2. Assay conditions and their implications for the equilibrium shift argument
The steady-state kinetics assays are performed at 500 nM HCMV Pr in 10% glycerol (lines 322–324), while the IC50 assays use 500 nM HCMV Pr in 5% glycerol (line 347). The dimerization Kd is reported as ~70 μM in saline and ~5.8 μM in 10% glycerol (lines 115–116). Even under the most favorable glycerol-stabilizing conditions, 500 nM is approximately 10-fold below the dimer Kd, meaning that the enzyme is already predominantly monomeric and thus largely inactive in both assays. The small fraction of dimer present at equilibrium is what provides the observed activity.
This has two important implications. First, the manuscript's comparison of Fab5 Kd (1.6 μM) to a "44-fold lower" dimerization Kd (Fig. 4d legend, line 680) uses the saline Kd of 70

μM. However, the steady-state kinetics assays are performed in 10% glycerol buffer where the dimer Kd is ~5.8 μM. Under these conditions the affinity advantage is only ~3.6-fold, substantially weakening the thermodynamic argument. Second, the IC50 assay uses 5% glycerol, where the dimer Kd is presumably intermediate between 5.8 and 70 μM but is not reported. The manuscript should explicitly reconcile which dimerization Kd applies under which assay conditions and present the equilibrium shift argument using condition-matched values.
I recommend a brief discussion acknowledging that at the assay concentration of 500 nM, the enzyme is predominantly monomeric under all tested buffer conditions, and clarify the buffer-dependent dimerization Kd values used in the thermodynamic comparison. The 44-fold claim in Fig. 4d should either use the condition-matched Kd or explicitly note that it refers to saline conditions. This is a textual clarification, not a request for new experiments.

Response to comment 2: We agree with all sub-points in this comment and have revised the manuscript accordingly.

We have revised the manuscript carefully to ensure all affinity comparisons are condition-matched. The Fab5 K_d (1.6 μM) was measured by BLI in the absence of glycerol, which is the same condition as the saline dimerization K_d of HCMV Pr (70 μM), making the 44-fold comparison internally consistent. Under steady-state kinetics assay conditions (10% glycerol, dimerization K_d of ~5.8 μM), the affinity advantage is 3.6-fold as the reviewer states. The IC_{50} assay has a final glycerol concentration of 5%, where the dimerization K_d is assumed to be between 5.8 and 70 μM, but is not measured. Our revisions are listed below.

Regarding the predominantly monomeric enzyme at 500 nM: We added a discussion in the results acknowledging this. We note that substrate binding has been shown to promote HCMV Pr dimerization[2]. While HCMV Pr is likely predominantly monomeric at the start of the assay, especially with a pre-incubation with Fab5, addition of the substrate should theoretically shift the equilibrium towards the dimer (although by how much, we are not sure). The fact that we observe mixed-type inhibition under these assay conditions in a similar manner to dimer-disrupting small molecule inhibitors we have previously reported supports the model that Fab5 sequesters HCMV Pr monomers.

We have made the following changes:

1) Figure 4d legend now reads "Fab5 binds HCMV Pr monomers with higher affinity than the dimerization threshold under all tested buffer conditions, shifting the equilibrium away from dimer formation and towards the Fab5-HCMV Pr complex."
2) Closing paragraph of the results section now reads: "These results demonstrate that Fab5 selectively binds and isolates HCMV Pr monomers. The Fab5 binding affinity (Kd= 1.6 μM, measured by BLI in the absence of glycerol) is approximately 44-fold higher than the dimerization Kd of HCMV Pr under matched buffer conditions (~70

μM in saline buffer), and remains favorable in 10% glycerol (~3.6-fold higher, vs. dimerization Kd of ~5.8 μM), which is the condition used for steady-state kinetics assays."

3) BLI methods subsection states that assays were performed without glycerol
4) IC50 methods subsection states that "The combination of HCMV Pr solution in 10% glycerol and Fab solution in PBS yields a final glycerol concentration of approximately 5%."

3. Causality of the latch–dimerization relationship requires more cautious framing
The data convincingly show that latch motif mutations (E122A, Y253A, K255A) abolish both activity and dimerization. However, this establishes correlation, not directionality. It is not demonstrated whether latch engagement drives the disorder-to-order transition of the C-terminus and thereby initiates dimerization, or whether dimerization occurs first and the latch locks the C-terminus in place afterwards. No structural intermediate of a pre-dimer, latch-closed monomer has been observed.
The Discussion appropriately refers to a "bidirectional relationship" (line 220). However, the Results section uses directional and causal language. The section heading "The Latch Loop Governs HCMV Pr Dimerization and Activity" (line 159) implies directionality. Lines 137–138 state that "disrupting the interaction between the C-terminal tail and the latch loop prevents the entire C-terminal region from folding," and lines 155–157 state "Disrupting the latch-tail interface prevents the C-terminal region from adopting its ordered form, revealing a previously unknown mechanism." This causal framing in the Results is not consistent with the appropriately hedged bidirectional model in the Discussion.
I suggest moderating the causal language in the Results to match the Discussion's bidirectional framing.

<u>Response to comment 3</u>: We agree that the causal language in the Results section should be brought into alignment with the bidirectional framing used in the discussion. We believe that the latch and C-terminal tail likely stabilize each other's ordered states cooperatively, and our data do not resolve the directionality of this relationship. The revisions listed below ensure that the results section aligns with the language used in the Discussion.

We made the following changes:

1) Section heading line 168: changed from "The Latch Loop Governs HCMV Pr Dimerization and Activity" to "The Latch Loop Is Essential for HCMV Pr Dimerization and Activity."
2) Line 146: changed from "This suggests that disrupting the interaction between the C-terminal tail and the latch loop prevents the entire C-terminal region from folding into its ordered, catalytically active form." To "This suggests that disrupting the

interaction between the C-terminal tail and the latch loop is associated with loss of ordered density for the entire C-terminal region."

3) Line 165: changed from "Disrupting the latch-tail interface prevents the C-terminal region from adopting its ordered form, revealing a previously unknown mechanism by which this conformational change required for activity is maintained." To "Disrupting the latch-tail interface is associated with loss of the ordered C-terminal conformation, consistent with a structural relationship in which the latch loop and C-terminal tail stabilize each other's ordered states. This reveals a previously uncharacterized structural element required for the conformational transition associated with HCMV Pr activation."

4. Interpretation of absent α5/α6 density and the OHL displacement
The authors state that "no EM density corresponding to [α5 and α6] helices appears even at low thresholds" (lines 136–137) and write that "This suggests that disrupting the interaction between the C-terminal tail and the latch loop prevents the entire C-terminal region from folding" (lines 137–139). I appreciate the use of "suggests" here. However, it is well established that the HCMV Pr monomer has a disordered C-terminus. Since the Fab5 complex is monomeric by definition, the absence of α5/α6 density is expected for any monomeric species and does not independently demonstrate that latch disruption is the cause of C-terminal disorder. The observation is consistent with the model but the later passage at lines 155–157, which states that "Disrupting the latch-tail interface prevents the C-terminal region from adopting its ordered form, revealing a previously unknown mechanism," reverts to causal language that is stronger than the underlying data support. Regarding the OHL, the authors appropriately note that they "can infer the orientation of the OHL from low-resolution density" (line 143) and that the OHL "appears to open by 5.6Å" (line 144). I appreciate the hedging language. However, the local resolution map in Fig. S6B indicates that the active site region falls in the 4–5 Å range. A precise measurement of 5.6 Å from a region at this resolution may convey more confidence than warranted. Reporting this as "approximately 5–6 Å" or "density consistent with an open OHL conformation" would better reflect the underlying data quality.
I recommend reframing lines 155–157 to be consistent with the "suggests" language used at lines 137–139. Consider softening the OHL displacement to an approximate range rather than a precise value.

<u>Response to comment 4</u>: We agree with both points and have revised the relevant sections accordingly.

Regarding the absent α5/α6 density: The reviewer is correct that the HCMV Pr monomer has a disordered C-terminus by default, and that the absence of this density in its monomeric form is expected. This observation is consistent with our model but does not on its own demonstrate causality. We revised the results section to use language

consistent with the “suggests” framing used elsewhere in the manuscript and to make clear that structural observations are made in the context of the full body of evidence presented.

Regarding the OHL displacement: We agree that reporting a precise 5.6Å value for the displacement in a region with lower local resolution conveys more confidence than the data warrants. We have revised the text as the reviewer suggests.

1) Line 144: changed from “The OHL appears to open by 5.6Å (measured from the α-carbon of R165)” to “The OHL appears to be displaced by approximately 6 Å relative to the active dimer conformation (measured from the α-carbon of R165), consistent with an open OHL conformation ….”
2) Figure 2h legend and figure: Updated with the more approximate OHL displacement measurements.

5. K255A thermal destabilization and DSF concentration discrepancy
The DSF data (Fig. 3d) show that K255A exhibits a Tm of 44.85 ± 0.09°C versus 48.28 ± 0.05°C for wild type, a decrease of 3.4°C. The figure legend (line 670), however, states the decrease is “4°C.” This rounding discrepancy should be corrected for consistency.
More substantively, there is a discrepancy in the reported DSF protein concentration: the Methods section states “Protein (2 μM)” (line 394), whereas the Results state that experiments were “performed at an HCMV Pr concentration of 4μM” (line 176). This should be corrected.
Regarding interpretation, a 3.4°C Tm decrease is not negligible and raises the question of whether K255A’s loss of activity is partly attributable to local destabilization of the C-terminal region rather than exclusively to disruption of the latch–tail interaction. The authors state that these results “indicate that the latch mutations do not substantially compromise the overall protein fold or stability” (lines 180–181), but the K255A Tm shift is notably larger than the other mutants (≤1°C) and deserves individual comment.
Additionally, DSF at the reported concentrations (2 or 4 μM) is well below the dimerization Kd, meaning it primarily measures monomer thermal stability and does not address whether K255A specifically destabilizes the dimer interface.
I recommend correcting the concentration discrepancy between Methods and Results, correct the rounding in the figure legend, and add a sentence noting the K255A Tm shift as a caveat. Acknowledge that DSF at sub-Kd concentrations primarily reports on monomer stability.

Our response to comment 5: We thank the reviewer for catching the discrepancy. We have corrected the figure legend to report the K255A Tm decrease as 3.4°C. We have also reconciled the DSF protein concentration between the methods and results sections.

Regarding the Tm shift: The reviewer makes a fair point that a 3.4°C decrease is notably larger than the other shifts observed. We added a sentence acknowledging that K255A exhibits a more substantial reduction in apparent melting temperature, noting that this raises the possibility of local destabilization of the C-terminal region contributing to the loss of activity in addition to the latch-tail interaction. We also acknowledge that DSF performed at concentrations well below the dimerization Kd primarily reports on monomer stability.

1) Figure 3d legend: changed "Thermal stability profiles of HCMV Pr mutants assessed by DSF. The K255A mutation decreases thermal stability by 4°C compared to wild-type" to "Thermal stability profiles of HCMV Pr mutants assessed by DSF. The K255A mutation decreases thermal stability by 3.4°C compared to wild-type."
2) DSF methods subsection: Reconciled protein concentrations to all state 4 μM.
3) Added two sentences to the results section after line 198 acknowledging the larger K255A Tm shift and that sub-Kd DSF measurements primarily report on monomer stability.

Reviewer #2 (Remarks to the Author):

I thank the authors for their careful and thoughtful responses to my previous comments. The revised manuscript has been substantially improved in clarity, scope, and conceptual framing. The authors have appropriately tempered several claims, strengthened the discussion of mechanism, and clarified the limits of their conclusions.
Regarding the Fab5 binding mechanism, the expanded thermodynamic explanation of the monomer–dimer equilibrium and the inclusion of affinity comparisons (Fab5–HCMV Pr vs. HCMV Pr dimerization) significantly strengthen the argument. I also appreciate the more cautious positioning of the cryo-EM strategy as a proof-of-principle rather than a universally applicable method. The revisions to the Discussion appropriately reflect the case-dependent nature of extending this approach to other herpesvirus proteases. Importantly, the authors have more clearly articulated what is conceptually novel relative to prior structural studies on herpesvirus proteases, namely the identification of the latch loop as a regulatory element that stabilizes the C-terminal disorder-to-order transition during dimerization, and the use of an antibody as a conformational probe to trap the monomeric state. This framing now makes the advance over previous work clear.

<u>Response</u>: We sincerely thank the Reviewer for their thorough engagement with our manuscript across both rounds of review. Their comments in the first round improved both the clarity and scope of the manuscript, and we are pleased that our revisions addressed their concerns.

Reviewer #3 (Remarks to the Author):

Zimanyi et. al. have responded with an updated version of their manuscript. The

conclusions remain well-founded and the story interesting; however, I still have concerns about how the authors have addressed model building into low-density regions of the cryo-EM map.

Major comments:
1. The authors have clarified their model-building approach, but the issue of building a model into low-density regions of the cryo-EM map remains. There is not enough density in any of the three maps to build the distal parts of the protease, and the parts of the atomic model that include these regions are not supported by the cryo-EM data. Docking the existing crystal structure (as an unmodified atomic model) into the cryo-EM density, to show how the protease is oriented and where the loops likely are, is perfectly acceptable. Using the crystal structure as a starting point in model refinement into the cryo-EM density is also widely accepted. The problem is that the newly generated models in this manuscript were not truncated where the cryo-EM density is insufficient to accurately place residues. They will not have a structure identical to the one defined by x-ray crystallography, and being unable to see the distal parts of the protease at 4Å or better resolution means it is not possible to accurately place those regions of the protein in an atomic model.

If the depiction of the map in Fig. S6 is misleading and sufficient cryo-EM density exists to build the models in Fig. S6 and Table S1 are present, I would request to see the PDB/EMDB validation report and the Phenix refinement map/model correlation coefficient graph for the refined map/model combinations. If sufficient density is not present to support residue placement, the newly generated atomic models should be truncated to include only residues the cryo-EM density can support.

Response: We thank the reviewer for their attention to our models and the points made above. We have made the following changes to the manuscript and the deposited atomic models:

We have updated our models and re-deposited them in the PDB with a more rigorous treatment of model confidence. The updated models only contain side chains where the cryo-EM density clearly supports their placement. Where the cryo-EM density is present but insufficient for side chain placement, backbone coordinates are retained as a reference scaffold with side chains removed. These backbone-only regions correspond to the beta-barrel core of HCMV Pr, which has been shown to be structurally rigid, and their coordinates closely reflect the crystal structure in PDB:1CMV[3]. Residues lacking density support have been omitted entirely from the atomic models. We have updated our methods section to reflect this rationale as well.

We have revised Fig. 2h to only show the backbone of the OHL position, reflecting the 4-5 Å local resolution in this region. Fig. S6 has been revised to show local resolution estimates for all 3 volumes at two thresholds each, along with depictions of our updated atomic models where side chain atoms are depicted as spheres and backbone-only reference scaffold is depicted as cartoons. Table S1 has been updated with revised validation

statistics, and official validation reports are provided from the updated PDB depositions. We include our new models with the resubmission, along with a re-upload of our volumes.

Citations:

(1) Shahian, T.; Lee, G. M.; Lazic, A.; Arnold, L. A.; Velusamy, P.; Roels, C. M.; Guy, R. K.; Craik, C. S. Inhibition of a Viral Enzyme by a Small-Molecule Dimer Disruptor. *Nat. Chem. Biol.* **2009**, *5* (9), 640–646. https://doi.org/10.1038/nchembio.192.

(2) Marnett, A. B.; Nomura, A. M.; Shimba, N.; Ortiz de Montellano, P. R.; Craik, C. S. Communication between the Active Sites and Dimer Interface of a Herpesvirus Protease Revealed by a Transition-State Inhibitor. *Proc. Natl. Acad. Sci. U. S. A.* **2004**, *101* (18), 6870–6875. https://doi.org/10.1073/pnas.0401613101.

(3) Lee, G. M.; Shahian, T.; Baharuddin, A.; Gable, J. E.; Craik, C. S. Enzyme Inhibition by Allosteric Capture of an Inactive Conformation. *J. Mol. Biol.* **2011**, *411* (5), 999–1016. https://doi.org/10.1016/j.jmb.2011.06.032.

Zimanyi et. al. investigate the structure and function of the human cytomegalovirus (HCMV) serine protease encoded by UL80. The protease is essential for viral capsid maturation, and is a druggable target for both HCMV and related herpesviruses. To investigate the protease, the authors first screened a phage display library for Fab fragments that bound the protease. One of those Fabs, termed Fab5, also disrupted the protease's catalytic activity. To determine the mechanism behind Fab5's disruption of the protease, the authors then solved a cryo-EM structure of the Fab5/UL80 protease complex. In the structure, Fab5 interacts with residues 111-117 of the protease, preventing the protease's C-terminal tail from forming a dimeric interface and assembling into the dimeric, active form of the protein. The authors validate the cryo-EM model by mutating residues involved in the latch loop/C-terminal tail interaction and find both that these mutants fail to dimerize and that they lose their catalytic activity. The authors also note that the overall structure of herpesvirus serine proteases are well-conserved between the human herpesviruses, and demonstrate that corresponding mutations to the KSHV serine protease completely abolish its catalytic activity.

Overall, the findings are exciting, with clear implications for structure-guided drug design. The paper is also well written, with data that support the authors' conclusions. However, one major critique is that not all of the cryo-EM map is at the reported resolution of ~2.7Å, and large portions of the density (most corresponding to parts of the viral protease away from the antibody binding site) are at too low a resolution to support accurate atomic model building. Obtaining a cryo-EM structure of an ~70kDa complex is impressive, but the limitations of the cryo-EM map and the corresponding atomic model need to be clearer.

Major comments:

1. Given the small size of the protein complex (~70kDa), obtaining a map with a resolution suitable for building an atomic model is an impressive feat. Both the antibody and the latch loop are clearly at a density appropriate to build an atomic model. However, while portions of the three cryo-EM maps in the paper have density consistent with the reported resolution of ~2.7Å, other regions are at substantially lower resolution. Because of the lower resolution (below 4Å) and the inability to trace the peptide backbone or accurately place side chains, I do not think that it is appropriate to build an atomic model into these low-density regions. Rigid-body docking of the available UL80 crystal structure into the density, truncating chains when the map drops below ~4Å resolution, and/or color-coding low-resolution density to track movements instead of building into it, would all give a more accurate representation of the data. It would also be appropriate to include a local resolution map to illustrate where these regions of lower resolution are.

2. In addition to regions with lower resolution, all three maps demonstrate a substantial amount of over-fitting, evidenced by noise/spikiness in the maps even at moderate contour levels. This may be the result of 3D refinement using a mask that was too tight.

3. In the analysis of enzyme kinetics in Figure S3c, fitting of the Lineweaver-Burke plot at 1.5μM of Fab5 is poor. Is there a reason why the datapoint with the highest substrate concentration was such an outlier?

Minor comments:

1. In figure S4, it is difficult to determine what loops belong to what protein or where the catalytic triad is in S4b and S4c. Because of the shading, in 4b and 4c, it is not clear that two protein structures are superimposed. In S4a, having a protein loop that is both green and orange is also difficult to interpret. Labels or a key within the figure (rather than just the legend) could also improve clarity.
